# Drug and Clinical Candidate Drug Data in ChEMBL

**DOI:** 10.1021/acs.jmedchem.5c00920

**Published:** 2025-09-19

**Authors:** Fiona M. I. Hunter, Harris Ioannidis, A. Patrícia Bento, Nicolas Bosc, Sybilla Corbett, Eloy Felix, M. Paula Magarinos, Emma Manners, Ines A. Smit, Marleen de Veij, Noel M. O’Boyle, Barbara Zdrazil, Andrew R. Leach

**Affiliations:** European Bioinformatics Institute (EMBL-EBI), Wellcome Genome Campus, Hinxton, Cambridge CB10 1SD, United Kingdom

## Abstract

ChEMBL is a large-scale, open-access, FAIR database of bioactive molecules with drug-like properties. ChEMBL 35 contains 17,500 approved drugs, and drugs that are progressing through the clinical development pipeline. Drug curation has formed an integral part of the core offering of the ChEMBL database since its inception. The paper is a reference guide to present the principles of why the ChEMBL drug data has been curated in a particular manner so that data users can better understand the nature of the data. The drug data include information on: names, synonyms and trade names, chemical structure or biological sequence, data sources, indications, mechanisms, warnings and drug properties such as maximum phase of development, type of molecule, prodrug status and first approval. The integrated nature of the drug data within the context of a bioactivity resource enables the wide use of the data set in drug discovery, AI and machine learning.

## Graphical abstract

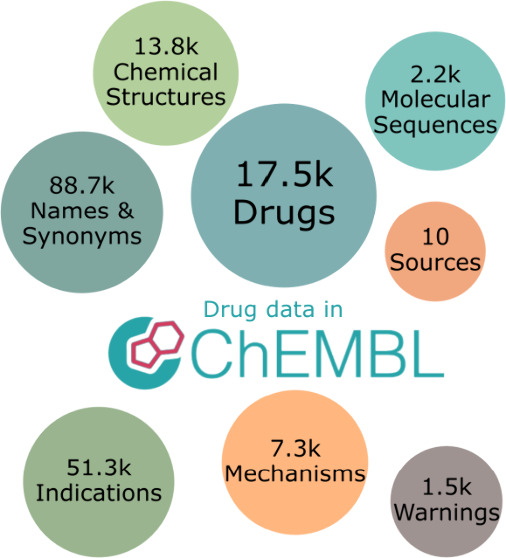

## Significance

- The paper presents the state-of-the-art processes to curate and integrate the high-quality drug and clinical candidate drug data in ChEMBL.
- It educates ChEMBL users, helping them to understand the nature of the drug and clinical candidate data and the rationale that underlies curation decisions.
- Given the increasing reliance on high-quality data in computational drug discovery, AI and machine learning, the integrated nature of the drug data within the ChEMBL bioactivity resource is a critical asset.

## Introduction

The first public launch of the ChEMBL database (www.ebi.ac.uk/chembl) in 2009 was a milestone in the recent history of chemical biology and drug discovery because it provided unprecedented free access to large amounts of high-quality, curated data on bioactive molecules. ChEMBL^1,2^ has grown significantly since then and now impacts a wide range of areas that include drug discovery, data science and the development and validation of AI, machine learning and other *in silico* methods (e.g., see references in ref 1). In 2022, in view of its fundamental importance to the biological and biomedical data community, ChEMBL was recognized by the Global Biodata Coalition^3^ as a Global Core Biodata Resource.

The inclusion of rich, high-quality, well-curated drug data within the structure of large-scale, open-access, FAIR^4^ database of bioactive molecules make ChEMBL an increasingly valuable and trusted chemical biology resource. It plays a central role within the landscape of open-access chemical biology resources as the scientific community moves toward wider use of artificial intelligence in drug discovery and its inherent dependence on high-quality data.^5^

Curation of data for approved drugs, and drugs that are progressing through the clinical development pipeline (“clinical candidate drugs”), has formed an integral part of the core offering of the ChEMBL database since its inception. The curated drug data enable the scientific community to answer important and practical science questions. Examples include the large-scale assessment of drug and ligand physicochemical properties and ligand efficiencies,^5^ the identification of drug repurposing opportunities,^6,7^ virtual screening for potential anti-SARS-CoV-2 drug candidates,^8^ identifying potential drugs to treat the neglected tropical disease, schistosomaisis,^9^ identifying drug targets for heart failure,^10^ investigating the reasons why clinical trials stop before their end points were met,^11^ or the use of drug indication data as a reference set to test emerging Large Language Models.^12^

Although a review of open access databases for drug data is beyond the scope of this article, it is worth mentioning some comparable chemical biology resources. For example, DrugCentral^13^ has been curating approved drug data since 2016 and contains good coverage of marketed drugs along with some bioactivity data (of which half comes from ChEMBL). However, they do not curate clinical candidate drug data, unlike ChEMBL. The Guide to PHARMACOLOGY^14^ contains ligand-activity-target relationships with a primary focus on target data rather than drug or clinical candidate drug data. Relevant approved and clinical candidate drugs are included for cases where e.g., the drug is bound to a newly identified target. PubChem^15^ is a widely used, open chemistry database whereby the data are contributed by many different depositors, but the data are not manually curated. Indeed, for each ChEMBL release, all chemical structures are deposited into PubChem. Open Targets^16^ aims to identify and prioritise therapeutic drug targets, and all its drug and clinical candidate drug data are sourced from ChEMBL. DrugBank^17^ contains comprehensive drug and clinical candidate drug data but does not provide this information within the framework of a bioactivity database for research compounds tested in experimental assays, unlike ChEMBL. In addition, DrugBank is only freely available for noncommercial use and therefore cannot be considered as a comparable open-access database. There are also subscription-based drug and clinical candidate drug data resources such as Pharmaprojects,^18^ or Cortellis Drug Discovery Intelligence,^19^ that are not open-access.

This manuscript presents the state-of-the-art processes to curate and integrate the high-quality drug and clinical candidate drug data in ChEMBL. It is educational in its approach and provides background detail for each database field that describes drug data. For each data principle, one or more examples are included so that the reader can easily understand the underlying logic that led to the curation of the data in a particular manner. Some of the examples explain complex approaches that have been taken, and the rules developed, to deal with tricky cases of curation where the outcome is not immediately obvious.

Drug and clinical candidate drug data were historically captured in a stand-alone, in-house database, and this approach has been retained to date. This means that all drugs, and drug-related data, are captured in our internal database, “Drugbase”, followed by periodic integration into the overall ChEMBL database. The consequence of this approach is that ChEMBL retains a clear distinction between manually curated drugs with annotated information, and compounds tested in assays with bioactivity data. This paper focuses on the curation of drug and clinical candidate drug data (Figure 1), although it is recognized that many of these drugs will also have associated bioactivity data in ChEMBL. The key areas of curation for drug and clinical candidate drug data include: the drug name, synonyms and trade names, chemical structure or biological sequences, data sources, drug indications, drug mechanisms, drug warnings. In addition, molecule features are captured for: maximum phase of development, dosed ingredient status, type of molecule, first-in-class flag, chirality, prodrug status, route of administration, availability type, therapeutic flag, first approval year for the marketed drug and year of clinical candidate application, orphan status (Figure 1).

**Figure 1:**
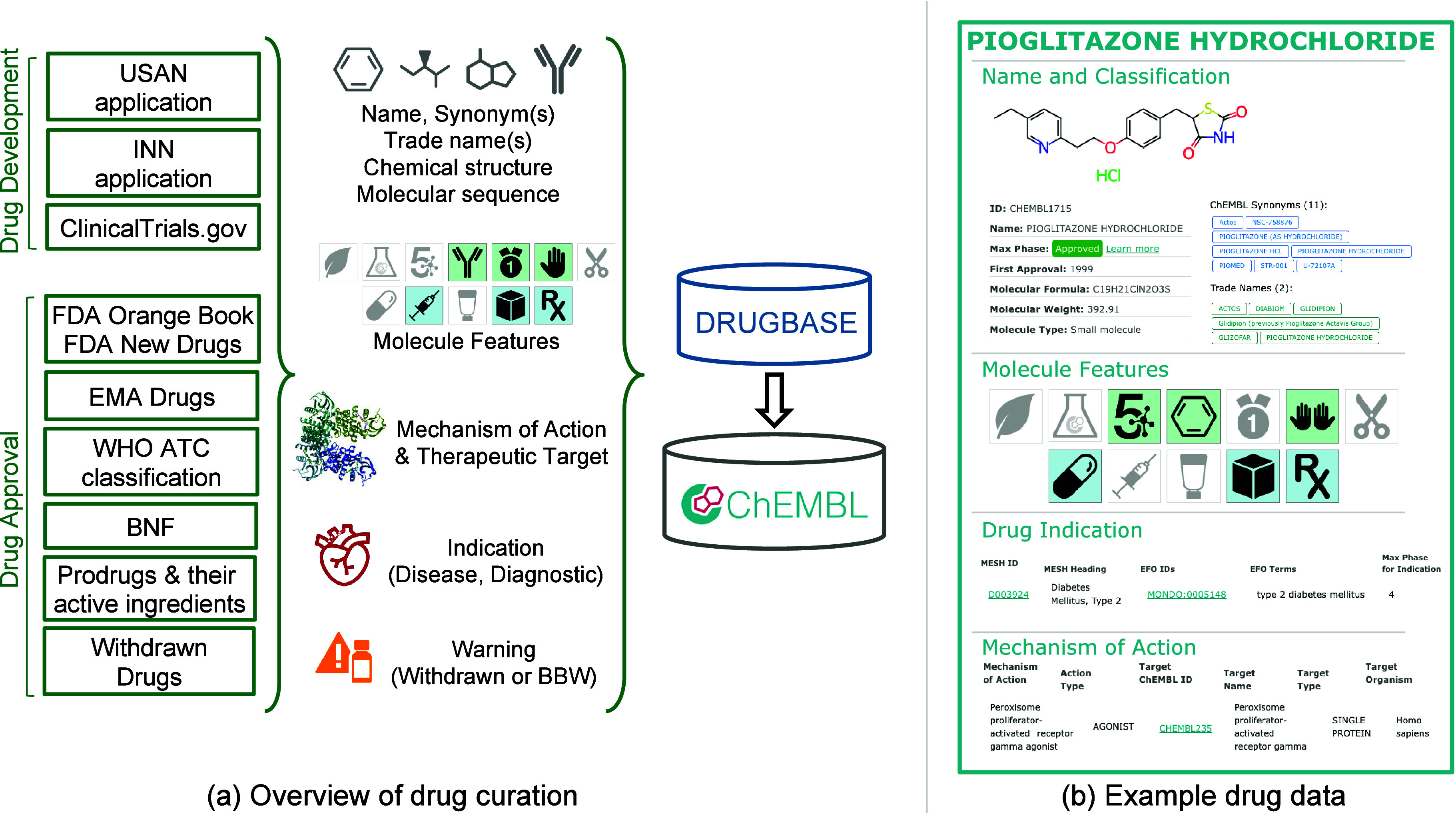
(a) Data for drugs and clinical candidate drugs in ChEMBL are curated from multiple sources of information. (b) Example of typical drug data for pioglitazone hydrochloride (CHEMBL1715).

ChEMBL 35 contains around 17.5 thousand marketed drugs and clinical candidate drugs (∼15.5 thousand parent drugs). In addition, there are around 2.5 million compounds with experimental bioactivity data (termed “research” compounds for the purposes of this paper). In essence, although a research compound must have associated bioactivity data, a drug or clinical candidate drug does not require experimental data for inclusion in ChEMBL. However, an approved drug could also be a clinical candidate drug and/or a research compound (e.g., pioglitazone hydrochloride, CHEMBL1715) and therefore it may also have additional attributes as shown in Table 1.

**Table 1:** How ChEMBL Distinguishes a Drug, a Clinical Candidate Drug, and a Research Compound with Experimental Bioactivity Data

ChEMBL 35	Defining feature for inclusion in ChEMBL	Typical features in ChEMBL	Occasional or absent features in ChEMBL
Research Compound (∼2.4 million)	Must have bioactivity data.	Usually measured in one or multiple assay(s) against one or multiple target(s).	Usually does not have a preferred name (*pref_name*).
		Usually comes from scientific literature, a data set that has been directly deposited into ChEMBL^20^ or patent information.	Sometimes has indication, safety warning or mechanism information (if also a drug).
Clinical Candidate Drug (∼14 k)	Must come from a source of clinical candidate information (e.g., USAN, INN, ClinicalTrials.gov).	Has a *pref_name*, usually a recognizable drug name or a research code.	Does not have safety warning data.
		Around 40% of clinical candidate drugs have bioactivity data. (i.e., it is also a research compound).	May have indication and mechanism information.
		May be a prodrug.	
Approved Drug (∼4 k)	Must come from a source of approved drug information (e.g., FDA, WHO ATC, EMA).	Has a *pref_name*, usually a recognizable drug name.	May have safety warning information (withdrawn status or black box warning).
		Around 70% of approved drugs have bioactivity data (i.e., it is also a research and/or clinical candidate compound).	May have metabolism data (for selected drugs).
		Usually has indication and mechanism information.	May be a prodrug.
			May have orphan status for rare diseases.

The multiple processes to extract and curate new drug and clinical candidate data, and integrate it with existing data, involve much manual and semiautomated curation. The aim of our curation is to provide the highest quality data which means that many checks are performed as part of these processes, and in cases where there are differences between various input data sources for an individual drug or clinical candidate drug, in-depth discussion between our curators may be needed before a conclusion can be agreed upon. The detailed nature of our drug curation processes is time-consuming and has meant that the drug and clinical candidate data are usually updated once per year (e.g., for ChEMBL 26, ChEMBL 28, ChEMBL 29, ChEMBL 30, ChEMBL 32, ChEMBL 34).

As far as possible, the original source of the drug and clinical candidate drug information is captured to maintain a transparent data audit trail for our data users. For example, the *indication_refs*, *warning_refs* and *mechanism_refs* tables store the primary references that our curators have annotated. In addition, there is an ongoing effort to check, update and include references for other drug data that currently do not have their primary references publicly exposed. Note that in this paper, all table names given in the downloadable ChEMBL database are shown in italics (e.g., *molecule_dictionary* table).

The manuscript includes novel approaches and significant advancements that have been developed for the drug and clinical candidate drug data. These fall into three broad areas for data quality, data transparency, and data integration and sustainable code, and are highlighted in the following paragraphs, with further detail given within the body of the manuscript.

## Novel Approaches to Data Quality

The ethos of ChEMBL is to provide the highest quality data. Drug data and clinical candidate drug data are curated from a range of disparate data sources that do not always agree, and therefore novel, rule-based data curation approaches have been developed for a variety of different cases. These rules have been built upon in-depth discussion by the data team, using examples of particular cases that are often not straightforward. The rules have been developed, clarified with example scenarios and formally written. They will serve as the basis for transparent and consistent data curation going forward but may need to be reviewed and adapted as future scenarios contain additional diversity of data. Rules have been developed for the maximum phase of development (see Maximum Phase of Development), assignment of the preferred name (see Preferred Name, Synonym and Trade Name Curation), the method for assigning the therapeutic flag (see Therapeutic Flag) and withdrawn drug curation (see Drug Withdrawal and Warning Information) while discussion is ongoing for rules regarding first-in-class curation (see First In Class). Broadening data coverage falls under the umbrella of data quality, and the drug and clinical candidate drug curation approach aims to capture as much information from each source of data. For example, the annotation of disease and diagnostic indications is captured not only from regulatory approval of drugs, but also from the “condition” listed for a Clinical Trial, the therapeutic area described within a USAN and/or INN application, and the indication captured by the levels of WHO ATC classification. The manual aspect of our data curation processes means that new technologies are easily identified by our curators and can be captured using additional categories, new data fields, or similar, if there is expected to be sufficient value to our data users. For example, additional categories have recently been added for the antibody drug conjugate type of molecule, new action type categories that link a drug to its therapeutic target for gene and protein therapies, and new data fields for orphan drugs, or veterinary drugs (planned). Recent work has seen a significant increase in the number and coverage of data checks incorporated as part of the data integration processes. For example, the inclusion of new sources of data for INN applications and EMA approved drugs has led to additional cross-checking and verification processes for differences in chemical structure extracted from different sources that use the same trade name or synonym (see Data Quality Assessment).

## Significant Enhancements for Data Transparency

Data transparency is a critical aspect of the FAIRness guidelines, and the drug and clinical candidate drug data has been enhanced with this in mind. Our curation processes now place a key emphasis on mapping the drug form that is specified in the underlying source information. For example, any annotation (such as a disease indication, therapeutic mechanism of action, safety annotation) is mapped to an individual drug form, and not to e.g., the parent drug form. The reference for the annotation (e.g., a web URL or scientific literature paper) should cite the individual drug form to maintain a clear link to the source information and thereby show the data provenance. Additional cross-references for the original source of the data have been provided for EMA, FDA, USAN and INN compounds, which is especially helpful in cases where the compound source does not include a InChI representation and therefore cannot be cross-referenced using e.g. UniChem (see Cross References). Documenting the date of data extraction, as well as its source and subsequent processing, is an important aspect of data provenance. Therefore, the extraction dates for our clinical trials and black box warning pipelines have been included in recent ChEMBL release notes with the aim of extending this to other drug and clinical candidate drug sources for future releases (see Data Sources).

## Significant Enhancements for Data Integration and Sustainable Code

The Clinical Trials Pipeline (see Clinical Trials Pipeline) was first developed in 2016 to accurately extract relevant interventions (i.e., clinical candidate drugs) and conditions (i.e., disease or diagnostic indications) from ClinicalTrials.gov and map these to entities in ChEMBL. This set of semiautomated processes combined with extensive manual curation has provided a key source of critical information for registering new clinical candidate drugs and associated information in ChEMBL, and this type of information is not available in any other comparable, open-access chemical biology resource. Compounds observed in Clinical Trials currently form half of all drugs or clinical candidate drugs in ChEMBL. The EMA pipeline for approved drugs was created in 2023. The EMA data are extracted from multiple pdf documents per medicinal product and webpage summary information. The lack of an overall data structure for the source of EMA data presents many challenges including, for example, accurately extracting and mapping the correct drug form, which may differ between the parent drug form shown in the summary information and the drug form shown in underlying documents. Therefore, the EMA pipeline uses a combination of semiautomated extraction and multiple checks with manual curation as required to maintain the high-quality result (see Data Sources). There has been a significant drive to improve the sustainability of our internal code base, documentation and working practices over the past few years, leading to significant improvement in our drug and clinical candidate drug processes (see Technical Process).

## Methods

This section describes the underlying concepts and rules that are used in the curation of drug and clinical candidate drug data in ChEMBL, with examples to explain the complexity of some of the data issues that may arise.

## Compound Registration

Compound registration is the process to determine whether an incoming compound is already stored in a database, and if not, registration is performed by assigning a new internal database identifier, the molecule registration number (“*molregno*”) in ChEMBL. This results in a unique set of compounds (nonredundant) which are stored in the *molecule_dictionary* table of ChEMBL. By contrast, the *compound_records* table provides all sources of data for each unique compound identifier, and therefore one *molregno* in the *compound_records* table may be assigned more than one “record_id” corresponding to each of the sources of data. Each unique *molregno* is assigned to at least one data source; this provides an easy way to identify the origin of the data for each compound (see Data Sources). For example ozanimod hydrochloride (CHEMBL3707246) has been assigned the unique *molregno* = 2039217, but the *compound_records* table shows that it has three data sources: FDA Orange Book, EMA and USAN.

First, validation and standardization of each incoming chemical structure is performed using the checker and standardizer modules of the ChEMBL Structure Pipeline^21^ that is built on the open source toolkit RDKit.^22^ Then, the compound registration system uses InChIKey (InChI v1.06 for ChEMBL 35) to compare against existing chemical structures within the database, which results in a match to an existing compound identifier (*molregno*) or the registration of a new compound identifier. For biotherapeutic compounds where the chemical structure is not available, a combination of automatically checking for an existing compound name and a manual checking process is used to map an incoming biotherapeutic compound onto an existing compound identifier, or to register a new compound identifier if no match is found. In some cases, biotherapeutic compound mapping is not straightforward because the complexities of different generations of biotherapeutics, or different manufacturing processes etc., mean that they may be distinct, e.g., von Willebrand factor human (CHEMBL4298126) is distinct from vonicog alfa (CHEMBL2107873) which is the recombinant form and has the synonym “von Willebrand factor (recombinant)”.

## Data Sources

ChEMBL aims to extract and update all drug and clinical candidate information from its primary source of information on a regular basis, usually annually. However, each primary data source uses its own individual schedule to include new data and make updates to existing data. As a result, the approach taken by ChEMBL is to use the most recent data available for each source at the time of the drug and clinical candidate data update. This means that the date that the information was extracted from each data source may vary over a period of up to several months for each ChEMBL release. Recently, the extraction dates for our clinical trials and black box warning pipelines have been included in the release notes,^23^ with the aim of extending this to selected drug sources for future releases.

There are currently ten data sources of drug and clinical candidate data in ChEMBL, and Table 2. Some of these drug sources (e.g., FDA Orange Book, WHO ATC, USANs, Clinical Candidates and FDA new drug approvals) have been available for many releases of ChEMBL, while other drug sources have recently been included to extend the coverage of drugs and clinical candidate drugs (e.g., EMA, INN).

**Table 2:** Drug and Clinical Candidate Drug Sources in ChEMBL

ChEMBL source identifier^a^	Source Description^b^	Short Name for Source^c^	Count of compounds^d^	First introduced^e^
-	ALL DRUG SOURCES	-	17501	-
8	Clinical Candidate Compounds	CANDIDATES	8875	CHEMBL 12 (2011)
9	FDA Orange Book Drugs	FDA_ORANGE_BOOK	2312	CHEMBL 9 (2011)
12	FDA Novel Drugs and Biotherapeutics	FDA_NEW_DRUGS	229	CHEMBL 12 (2011)
13	United States Adopted Names (USAN)	USAN	12590	CHEMBL 12 (2011)
36	Withdrawn Drugs	WITHDRAWN	224	CHEMBL 22 (2016)
41	WHO Anatomical Therapeutic Chemical (ATC) Classification of Drugs	ATC	3492	CHEMBL 9 (2011)
42	British National Formulary (BNF)*	BNF	1961	CHEMBL 23 (2017)
53	Pharmacologically Active Ingredient of a Prodrug	PRODRUG_ACTIVE	282	CHEMBL 29 (2021)
63	International Nonproprietary Names (INN) for Pharmaceutical Substances	INN	812	CHEMBL 32 (2023)
66	European Medicines Agency (EMA)	EMA	882	CHEMBL 34 (2024)

a The source identifier for the data, as assigned by ChEMBL (src_id).

b A brief description of the data source.

c The short name for the data source.

d The count of the number of distinct drug forms for each source for ChEMBL 35.

e The ChEMBL release and the year when the data source was first introduced. * The BNF source of drugs is no longer being updated.

For each data source, all relevant data are extracted (Figure 1). Typically, this would include the preferred drug name (*pref_name*), its synonyms (and any research codes or trade names) and chemical structure (for small molecules) or biological sequence (for biotherapeutic compounds), if available. The compound is mapped to an existing ChEMBL compound, or registered as a new compound within the database, as explained above. After the name and chemical structure/sequence information is mapped, further curation is performed for annotation of (i) indication, (ii) mechanism of action, (iii) safety data, and (iv) other drug attributes.

At each stage in the data extraction and loading process, our code uses an exact match of the chemical structure (or ingredient name if the chemical structure is not available in the primary data source) to automatically map and load as much information as possible. However, this process is augmented by multiple checks before each loading step that flag any inconsistencies so that all potential mismatches or ambiguities are manually inspected, discussed by the curation team, and corrected before loading into the ChEMBL database.

The FDA Orange Book database of Approved Drug Products with Therapeutic Equivalence Evaluations^24^ (FDA_ORANGE_BOOK, *src_id* = 9) is our main source of FDA approved drug products. The FDA Orange Book data files are downloaded,^25^ each Active Pharmaceutical Ingredient^26^ (called an “ingredient” for the remainder of the article) is mapped (by name or synonym) to an existing *molregno* or registered as a new *molregno*. For combination medicinal products where there is more than one ingredient, each ingredient is registered separately in ChEMBL. Any inconsistencies identified during compound mapping are investigated by manually inspecting the drug label and correcting as required. Compounds that are no longer available in the downloaded Orange Book data files have their source downgraded so that the Orange Book source for the compound in question is removed from the publicly released version of ChEMBL. The Orange Book products are loaded into the *products* and *formulations* tables, while the Orange Book patents are loaded into the *product_patents* table. The Orange Book patent use code^27^ information is also extracted and loaded into the *patent_use_code* table. At present, only FDA Orange Book information is available in the *products*, *formulations*, *patents* and *patent_use_codes* tables.

In addition to the FDA Orange Book data, recently approved FDA drugs (FDA_NEW_DRUGS, *src_id* = 12) are extracted, mapped and loaded. In a similar method to the FDA Orange Book compounds, a process is used to check and correct compound mapping inconsistencies and to amend any compounds that previously had a “FDA New Drugs” source but are now included in the “FDA Orange Book” source. All new FDA drug approvals are extracted from e.g.,^28^ as well as selected FDA Biological License Application approvals from e.g.,.^29^ The FDA updates these web pages on a regular basis throughout the year, which means that a ChEMBL release includes all recently approved FDA drug approvals at the time of data extraction and loading, but this may not necessarily contain all newly approved drugs for a complete calendar year. For example, if data loading took place in mid-November, then ChEMBL would include all new drug approvals up to mid-November, but not those that were approved in late November nor December of the most recent calendar year.

European Medicines Agency drug approvals^30^ (EMA, *src_id* = 66) for human and veterinary medicines were identified using their advanced search.^31^ At present, EMA represents the only source of veterinary medicines in ChEMBL that are extracted on a regular basis. Much of the EMA medicine data is contained in multiple pdf documents, and a significant manual curation effort has been undertaken to check and accurately map the correct drug form that is specified within the detail of their documentation. Typically, the “ANNEX I: SUMMARY OF PRODUCT CHARACTERISTICS” and/or “ANNEX III: LABELLING AND PACKAGE LEAFLET” in the EMA Product Information (e.g.,^32^) is consulted to ascertain whether the ingredient is described as the salt form (e.g., imatinib mesylate - as given in the Product Information pdf) rather than the parent form (e.g., imatinib - as given by the summary information on the web page). Any ambiguities in the drug form of the medicinal product were further clarified using the EMA Public Assessment Report (e.g.,^33^), and in some cases the trade name was checked against information from other regulatory agencies (e.g., FDA^24^) or GSRS.^34,35^

The World Health Organisation (WHO) endorsed the Anatomical Therapeutic Chemical Classification (ATC) and Defined Daily Dose (DDD) methodology for global use in 1996 as the gold standard for drug utilization monitoring and research.^36^ The ATC classification has been adopted in various countries as a national standard for classification of medicinal products.^37^ The ATC classification system groups the ingredients according to the organ or system on which they act and according to their therapeutic, pharmacologic and chemical properties. The DDD is a unit of measurement and is linked to the ATC code.^38^ The ATC/DDD Index normally uses the INN name (which is typically the parent drug form) for the ingredient. For each ChEMBL release, all data in the ATC/DDD Index are extracted^39^ (ATC, *src_id* = 41). New ATC ingredients are mapped (by name or synonym) to an existing *molregno* or registered as a new *molregno* if the ingredient can be corroborated against an independent source of chemical structure or biological sequence information such as INN, USAN or GSRS. Some ATC ingredients may not be registered as a *molregno* because there is no unambiguous chemical structure or biological sequence information. For example, “estradiol, combinations”, ATC code = G03CA53 has not been registered. Similarly, the ATC combination “fluoxetine and psycholeptics”, ATC code = N06CA03) because “psycholeptics” has an ambiguous chemical structure even though fluoxetine (ATC code = N06AB03) is registered as CHEMBL41. Any obsolete ATC codes or descriptions are updated. Existing drugs with an ATC source in ChEMBL are checked and updated for the presence of additional ATC codes in the latest ATC/DDD Index.

The United States Adopted Names (USAN) Council selects simple, informative and unique nonproprietary (generic) drug names.^40^ A USAN name is usually applied for when the compound is in Phase I or Phase II clinical trials.^41^ All USAN compounds are published via the USP Dictionary of USAN and International Drug Names^42^ or, for those USAN applications that were granted after 1992, are available using the USAN search Web site.^43^ USAN compounds are manually extracted and nearly 200 hundred new USAN compounds were registered for ChEMBL 34 (USAN, *src_id* = 13). The typical information curated includes the USAN name, synonym(s), trade name(s), chemical structure, biological sequence (if appropriate) and therapeutic area.

The WHO, in collaboration with experts, selects a globally recognized, unique name for each active substance that is to be marketed as a pharmaceutical.^44^ These are published biannually via the International Nonproprietary Names (INN) program. The INN name is usually presented as the parent drug form. Other approved generic names are usually identical to the INN name, for example: British Approved Names (BAN), Dénominations Communes Françaises (DCF), Japanese Adopted Names (JAN) and United States Adopted Names (USAN). The INN name is usually applied for when the development of the drug has reached Phase II clinical trials.^45^ INN compounds are manually extracted from the latest proposed INN list^46^ and nearly 600 new INN compounds were registered for ChEMBL 34 (INN, *src_id* = 63). The typical information curated includes the INN name in English, French and Spanish, chemical structure, biological sequence (if appropriate), and therapeutic area. The proposed INN list, rather than the recommended INN list, is used as the source of the data so that the information is available to our data users at the earliest opportunity.

ClinicalTrials.gov is a Web site and database of clinical research studies.^47^ As a result of national laws and policies that have set the expectation that clinical trials should be listed in public databases, it contains much information about clinical trials that have been conducted worldwide. Our clinical trials pipeline extracts all relevant clinical trials and uses a curation process to map the intervention name to a *molregno* (CANDIDATES, *src_id* = 8) and map the clinical trial condition(s) to an indication. Additional information such as the development phase of the clinical trial is also extracted. More detail on the pipeline is provided in the Clinical Trials Pipeline section.

The British National Formulary (BNF, *src_id* = 42) contains information on the selection, prescribing, dispensing and administration of UK medicines.^48^ BNF data for medicine names and chemical structures (usually shown as the parent drug form) was extracted between 2017 (ChEMBL 23) and 2019 (ChEMBL 26). More recent updates to BNF have not been captured due to their amended licensing arrangements that prohibit the distribution of their data.

Withdrawn drugs are manually curated from information provided by national regulatory bodies, international agencies such as the WHO and the scientific literature (WITHDRAWN, *src_id* = 36). More detail is provided in the section entitled Drug Withdrawal and Warning Information.

Prodrugs and their pharmacologically active ingredients are manually curated from approved drug labels and the scientific literature. The pharmacologically active ingredient of a prodrug is assigned to the source PRODRUG_ACTIVE (*src_id* = 53) assuming that the prodrug itself is present in ChEMBL. More detail is provided in the section entitled Prodrugs and Drug Metabolism Data.

## Preferred Name, Synonym, and Trade Name Curation

For each drug and clinical candidate drug data source all relevant data are extracted. Typically, this would include the preferred drug name (*pref_name*), its synonyms and any research codes or trade names. For some sources, e.g., USAN or INN, the *pref_name* is normally clearly defined. However, other sources of drug information can be ambiguous and care is needed to accurately map the name of the drug form specified within the detail of the underlying source information, e.g., the salt form, abacavir sulfate (CHEMBL4303288), is described in the EMA product information,^49^ but their web summary information only describes the parent drug form, abacavir.^50^ In some cases, especially for biotherapeutic compounds that have not (yet) been assigned a USAN or INN name, or for older drugs or clinical candidates, multiple sources are consulted before a decision can be agreed between our curation team. As a result, we have developed some rules to help assign the *pref_name* used in ChEMBL:

1. If the compound is an approved drug, the drug name given by the regulator (EMA, FDA etc.) is assigned (this is usually the same as the GSRS preferred name).
2. If the compound is not an approved drug: (i) The USAN name is used in the first instance, (ii) If not available, then the INN name is used, or (iii) If USAN/INN are unclear or not available (e.g., for a research code) then the preferred name from GSRS is used. Note that Greek letters like “α” and “β” should be written out in full in English such as “alpha” or “beta”.

ChEMBL uses the concept of compound “families” so that each molecule has a parent compound (see Molecule Hierarchy). Historically, if the parent drug form was not available in its own right from a data source, the incoming chemical structure would be stripped of its salt(s) and solvent(s), and the same *pref_name* would be assigned for both the salt and its parent drug form. This legacy approach has meant that any incoming drug or clinical candidate drug would match by name (or synonym) to more than one ChEMBL compound, resulting in an additional curation burden to manually check and map to the correct drug form. Therefore, use of the recently developed *pref_name* rules helps our curators assign a distinct *pref_name* to each incoming drug form and its parent. However, some legacy cases remain where a single *pref_name* has been assigned more than one drug. Similarly, there are also legacy cases where a single synonym has been assigned to more than one drug. To help resolve these issues, there is an ongoing curation effort to clean up duplicate names or synonyms which will provide more clarity for future ChEMBL releases.

Trade names are included in the *molecule_synonyms* table if this is straightforward. For instance, the trade name is curated from USAN applications which clearly provide information on an individual drug form. However, for FDA Orange Book compounds, the approach taken by ChEMBL to prioritize the registration of single ingredient medicinal products means that FDA trade names where there is more than one ingredient (i.e., a combination medicinal product) have typically not been included in the *molecule_synonyms* table. The inclusion of a more comprehensive set of synonyms and trade names, including those for combination medicinal products, will be addressed for future ChEMBL releases.

## Molecule Structure

The chemical structure for drug data in ChEMBL is extracted from a range of types of source information. For some sources (e.g., USAN and INN) the chemical structure is always depicted, for other sources (e.g., FDA and EMA) the chemical structure is usually depicted in the underlying drug label information. However, some sources only provide a name, and do not provide a chemical structure (e.g., ATC, ClinicalTrials.gov). As a result, the approach used to extract and confirm the chemical structure depends on the source of the drug data.

SMILES (Simplified Molecular Input Line System)^51^ is an accessible and human-readable chemical line notation that encodes a chemical structure as a linear string of symbols that is similar to natural language. A canonical (unique) SMILES is generated from the V2000 molfile using RDKit (the current version is displayed in the version table, with RDKit version 2022.09.4 used for ChEMBL 35). InChI^52–54^ describes chemical substances in terms of layers of information: the atoms and their bond connectivity, tautomeric information, isotope information, stereochemistry and electronic charge. The InChIKey is a fixed length (27 character), condensed, digital representation of the InChI that is not designed to be human-understandable. The standard InChI and InChIKey are generated from the V2000 molfile using InChI (the current version is displayed in the version table, with version 1.06 used for ChEMBL 35). The resulting structural data (V2000 molfile, standard InChI, standard InChIKey, and canonical SMILES) are displayed in the *compound_structures* table.

ChEMBL uses V2000 molfiles to generate the standard InChIKey for its compound registration system. There are limitations of using this method and there is a very small chance that an InChIKey may not be unique (e.g., if the compound contains more than 12 ions that are charged). Therefore, ChEMBL is preparing for the change to V3000 molfiles in anticipation of the release of a method by the InChiTrust to convert V3000 molfiles to a standard InChI.

For USAN and INN compounds, our chemistry curator draws the chemical structure. The chemical structure is typically drawn from scratch because this provides a more accurate, time-efficient result than, for example, using an “image to molfile” recognition tool where each atom, bond, connectivity, chirality, charge etc. needs to be double checked. To maintain the highest quality chemical structure data, this approach can lead to further discussions about the exact nature (e.g., stereochemistry) of an individual structure with USAN or INN. The trickiest cases can include instances of multiple stereochemical centers e.g., ertugliflozin pidolate (CHEMBL5315054).

As described in Data Sources, the chemical structure for approved drugs is usually displayed in the medicinal product information such as the drug label (for FDA drugs), or the Summary of Product Characteristics (for EMA drugs). For newly extracted approved drugs, our curation team checks the chemical structure before it is loaded into the database. Any inconsistencies in existing approved drugs may be flagged up during our internal checking procedures, for example if two different sources of drug data show the same name, but different chemical structure (i.e., different InChIKey), or vice versa. Any chemical structure or name issues will be discussed internally and curated, before loading or updating ahead of the next ChEMBL release. Common issues include (i) the data source describing the compound parent name, although other sources of compound name show the salt, or hydrated form, (e.g., the imatinib mesylate example given earlier) or (ii) a difference in the stereochemistry between different sources despite an identical or similar compound name. For example, the USAN compound milnacipran hydrochloride (CHEMBL4297064) where the chemical structure has two chiral centers and is shown by USAN as two distinct stereoisomers in nonspecified proportions.^55^ Because the proportions of the two stereoisomers are not known, the stereobonds are not specified in ChEMBL, although the chemical structure is displayed. Compare this to the USAN compound, levomilnacipran hydrochloride, (CHEMBL2105732 ^56^) that also has two chiral centers but, given its absolute stereochemistry, it is depicted as the single enantiomer and both stereobonds are displayed.

Missing stereobonds or a partially described chemical structure given by the original source literature can lead to difficulties when merging drug and research compound sources of information. As far as possible, all chemical structures are extracted from the scientific literature without modification. However, there is a balance that sometimes requires additional manual curation of compounds (and also of targets). For example, lestaurtinib (CHEMBL603469) is a clinical candidate drug with a USAN source that clearly defines the chemical structure. A similar research compound has been extracted from the scientific literature but the stereochemistry is only partially defined in the corresponding article (CHEMBL175105, with the compound name “CEP701”^57^). Therefore, relevant bioactivity data for these distinct compounds is correctly split across the two compound entries in ChEMBL. In some cases, such compound entries can be merged based on the compound name provided in the scientific article, and the drug name or synonym from a known drug source, although this requires manual checking and curation.

For data sources where the chemical structure is not provided (e.g., ATC, ClinicalTrials.gov), the compound name is matched against (i) existing compound names and synonyms for drug sources in ChEMBL, (ii) existing compound names and synonyms for nondrug sources in ChEMBL and (iii) compound names and synonyms in GSRS, respectively. For each positive name match, the InChIKey is then back-mapped to an existing compound in ChEMBL to check for any inconsistencies in name or synonyms such as spelling or isotopic nomenclature differences etc. Any inconsistencies in name or InChIKey mismatches are sent to our curation team for discussion and curation. If correct, the chemical structure is checked, standardized and assigned to the incoming compound before loading as a new data source for an existing compound, or registered as a new compound.

The chemical structure is converted to a V2000 molfile format, and the ChEMBL Structure Pipeline^5^ is applied to check and standardize each structure. Only chemical structures with a penalty score of 5 or less are included. If the penalty score is greater than 5, the compound is flagged up to our chemistry curator for manual inspection and correction (if appropriate).

In some cases (namely, mixtures of compounds, polymers, organometallic complexes and biotherapeutic compounds), the chemical structure is not always displayed. This approach has been based on practical considerations where the detail of the chemical structure is not sufficiently known or cannot be accurately displayed. Further details are given in Table 3.

**Table 3:**
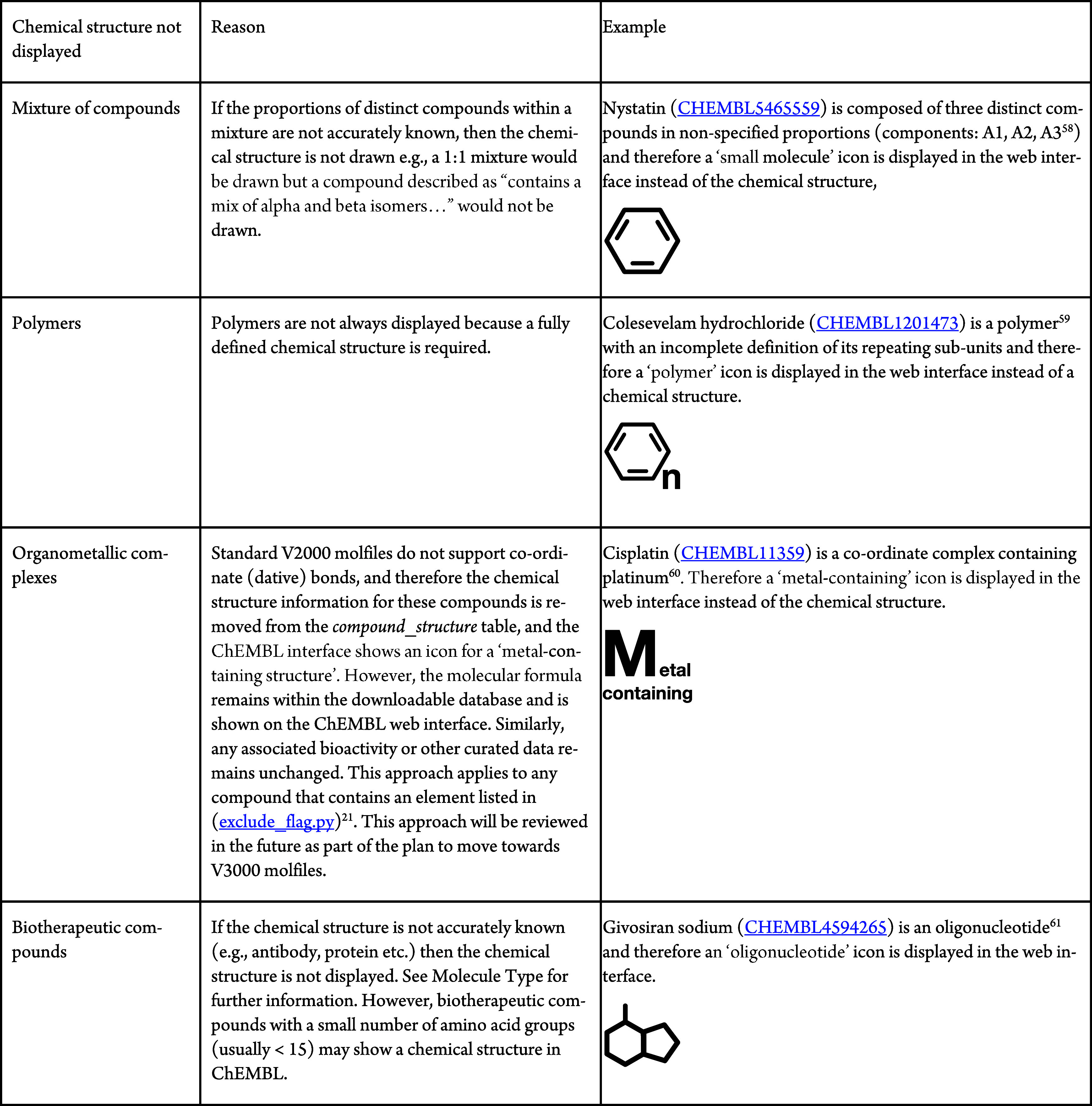
Examples of Cases Where the Chemical Structure Is Not Displayed in ChEMBL^58–61^

## Biological Sequence

Molecule sequence information is extracted for recent USAN and INN compound data, where available. For small biotherapeutic compounds, like peptides, usually both the chemical structure and the biological sequence are shown in ChEMBL e.g., maraciclatide (CHEMBL3989765) or vapreotide acetate (CHEMBL6067976). The biological sequence information given by USAN or INN is provided for proteins and nucleic acids. The information may include light or heavy chain protein sequences as well as the location of disulfide bridges, glycosylation sites etc. Similarly nucleic acid sequences may include additional information such as the sense or antisense strand. For example, lemzoparlimab (CHEMBL4650520, USAN source data, INN source data) contains a light and a heavy molecular sequence as well as post-translational modifications for the locations of disulfide bridges, N-terminal glycosylation sites and C-terminal lysine clipping. Currently, only the protein or nucleic acid sequence is available in ChEMBL, but we plan to include post-translational modifications, if appropriate, for future releases. For ChEMBL 35, there are 1324 distinct drug and clinical candidate compounds (as parents) with a protein sequence and 125 compounds with a nucleic acid sequence. The detailed sequence data are stored in the *bio_component_sequences* table while the *biotherapeutic_components* table is used to map to each compound identifier (*molregno*).

The HELM^62,63^ (Hierarchical Editing Language for Macromolecules) notation provides a representation of large and complex biomolecules (e.g., proteins, nucleotides, antibody drug conjugates) that are impractical to represent using existing small molecule or sequence-based informatic methods. The HELM monomer notation has been included for some peptide sequences. It was last updated for ChEMBL 25 (2018).

## Molecule Hierarchy

ChEMBL uses a hierarchy of compounds whereby any specific drug form belongs to a family of compound structures that contains one parent (a salt-stripped/solvent-stripped/isotope-stripped compound) and one or more salts/solvates/isotopes. The hierarchy can be accessed via the *molecule_hierarchy* table as follows (with examples shown in Figure 2):

- The *molregno* field is the unique identifier for a compound in ChEMBL. If the compound is an approved drug, the *molregno* field contains the drug form that is administered to the patient (e.g., the salt/solvate/isotope drug form). This is commonly called the Active Pharmaceutical Ingredient (API) by regulatory authorities.^26^
- The *parent_molregno* field contains the parent drug form which is the compound after any salt(s), solvent(s) and isotope(s) have been removed using the ChEMBL Structure Pipeline GetParent module.^21^ The use of the *parent_molregno* field allows all compounds within the same family to be easily identified or grouped. Compounds that are only generated by removing a salt/solvate/isotope and are not associated with any data in their own right (i.e., a virtual parent compound) appear in the *parent_molregno* field (and do not appear in the *molregno* field in the *molecule_hierarchy* table, and have no records in the *compound_records* table. Note that for a few compounds, the neutralization rules of the GetParent module^21^ may result in a tautomeric form of the parent compound that is not the most chemically stable. For these cases, the hierarchy between the *molregno* and *parent_molregno* is manually curated using an internal “*manually_fixed*” flag which overwrites the standard hierarchy and is not publicly exposed to our data users. For example, the generation of the parent compound of molidustat sodium (CHEMBL3931782) would result in the “enol” tautomeric form rather than the more stable “keto” tautomeric form (molidustat, en these compounds has been manually fixed. Similarly, for biotherapeutic compounds or mixtures that do not have a chemical structure in ChEMBL, this approach has been applied in some cases to link compounds within a family. For example, the *molecule_hierarchy* links the oligosaccharide muparfostat sodium (CHEMBL4297162) to its parent compound muparfostat (CHEMBL4297163). For ChEMBL 35, there are 31 drugs or clinical candidate drugs that have a manually curated *molecule_hierarchy*.
- The *active_molregno* field contains the compound that interacts with the target within the body to treat a disease or generate a phenotype. If the compound is a prodrug, the *active_molregno* will be different to the *parent_molregno*. More detail is given in Prodrugs and Drug Metabolism Data.

**Figure 2:**
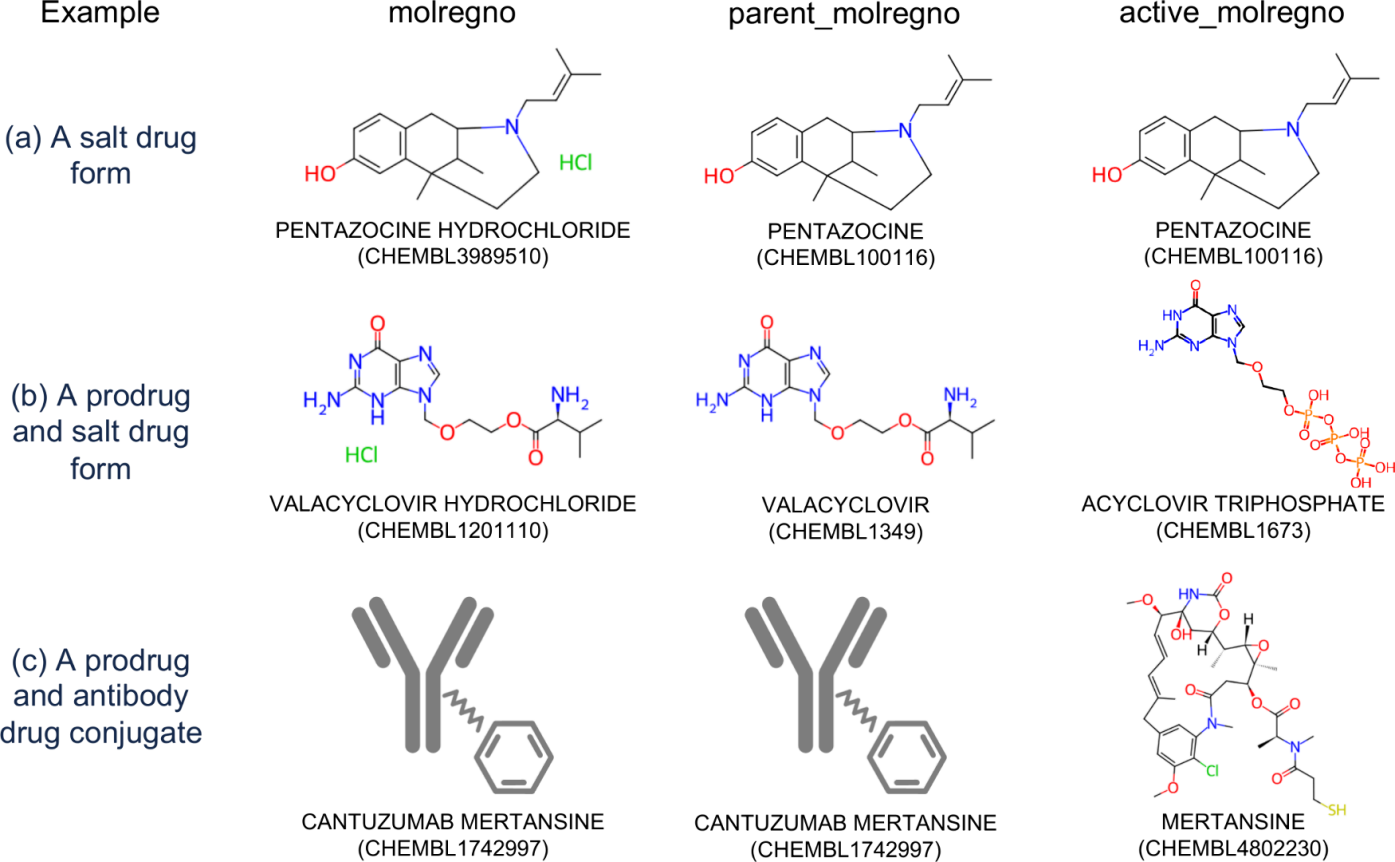
Examples and explanation of the fields in the *molecule_hierarchy* table. (a) The compound (pentazocine hydrochloride, an approved salt drug form) is the dosed ingredient and differs from the parent compound (pentazocine). The parent compound is also the *active_molregno* (i.e., not a prodrug). (b) The compound (valacyclovir hydrochloride, a prodrug and an approved salt drug form) is the dosed ingredient that differs from the parent compound (valacyclovir). The parent compound is metabolized within the human body to give the pharmacologically active ingredient (acyclovir triphosphate). (c) The compound (cantuzumab mertansine, a prodrug and an antibody drug conjugate in Phase II clinical trials) is the dosed ingredient and is also the parent compound. The compound is metabolized within the human body to give the pharmacologically active ingredient (mertansine) and the antibody component (cantuzumab) is the key to targeting the drug within a specific cell.

## Clinical Trials Pipeline

Our Clinical Trials Pipeline has been run on a regular basis since 2016 (typically 2 to 3 times per year). It extracts relevant data from “interventional” clinical trials, and for each clinical trial, it maps the intervention(s) that is being tested and the condition(s) that is being studied (Figure 3). The curated output from the pipeline is included within each drug update of ChEMBL. In addition, clinical trials for approved and clinical candidate drugs in ChEMBL are incorporated into the Open Targets Platform^16^ where the data are displayed under the heading of “Clinical Precedence”.

**Figure 3:**
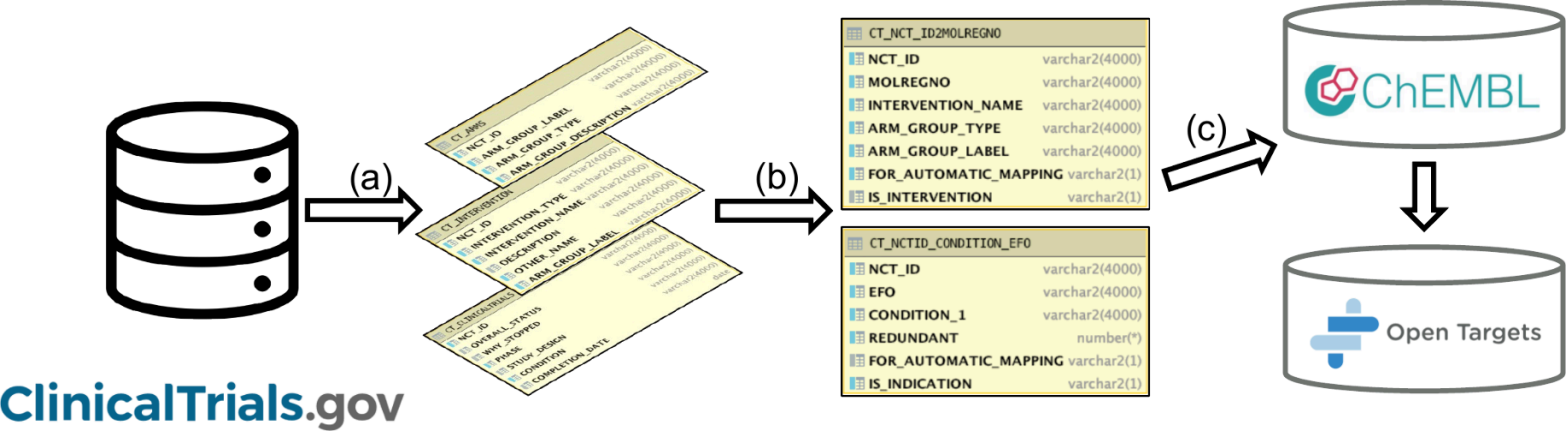
Clinical trials pipeline. (a) Relevant clinical trials are extracted from ClinicalTrials.gov and stored in our internal database staging tables. (b) For each clinical trial, the intervention(s) and condition(s) are mapped to a compound identifier (*molregno*) and a disease identifier (EFO id). (c) The mapped data for clinical trials are migrated to the ChEMBL database (via our internal “Drugbase” database) for public release and are also delivered to the Open Targets Platform.

## Extracting Data from ClinicalTrials.gov

Relevant clinical trials are extracted from ClinicalTrials.gov as follows (Figure 3a). Interventional clinical trials with a development phase assigned (i.e., the exact phase is described, not given as “Not applicable”), and a “last_update_posted” date within a specified time period are extracted. The time period specified in the “last_update_posted” field includes all data between the last date that our pipeline was run and the current date. An example API query that was used to access their data from 29th June 2023 onward is

https://www.clinicaltrials.gov/api/v2/studies?format=json&query.term=AREA%5BStudyType%5DInterventional+AND+%28AREA%5BPhase%5DEarly_phase1+OR+AREA%5BPhase%5DPhase1+OR+AREA%5BPhase%5DPhase2+OR+AREA%5BPhase%5DPhase3+OR+AREA%5BPhase%5DPhase4%29AREA­[LastUpdatePostDate]­RANGE­[2023-06-29,MAX]

This approach means that all new clinical trials are captured, as well as previously extracted clinical trials that have had any data updated, within the specified range of dates.

The extracted clinical trial data are stored in three internal database staging tables called *ct_clinicaltrials*, *ct_intervention* and *ct_arms*. These tables are not made publicly available. The *ct_clinicaltrials* table includes the unique Clinical Trials identifier of the trial (NCT), the overall status of the trial (e.g., “Completed”, “Recruiting” etc.), clinical development phase (e.g., Phase 4, Phase 3 etc.), study design, condition, and title of the trial. The *ct_arms* table includes the NCT identifier, arm group label, arm group type and arm group description. The *ct_intervention* table includes the NCT identifier, intervention type, intervention name and arm group label.

## Mapping the Intervention Name(s) and Condition(s)

A semiautomated curation process is applied to data extracted for each clinical trial (Figure 3b). In brief, the process extracts the intervention name(s) and maps this to a drug, and extracts the condition(s) that is being studied and maps this to an indication in the Experimental Factor Ontology (EFO). Any exact matches between the intervention name and a drug name are automatically assigned. Similarly, any exact matches between the condition that is being studied and an indication term in EFO are automatically assigned. Any nonexact matches (for either an intervention or a condition) are manually curated. Each iteration of the Clinical Trials Pipeline has provided the opportunity to extend our mapping dictionaries of intervention names and clinical trial conditions. For each of these curation processes, more explanation is provided below.

The following paragraphs describe the detail of the intervention mapping. For each clinical trial, the intervention name(s) is mapped (by name) to a drug form in ChEMBL and stored in an internal *ct_nct_id2molregno* mapping table that is not made publicly available. The *ct_nct_id2molregno* table also acts as a curated dictionary of each intervention name and its equivalent *molregno*. This is performed in a semiautomated manner whereby an exact match of the intervention name against a *pref_name* (or *synonym*) in ChEMBL is automatically mapped. However, any intervention name that does not exactly match a *pref_name* or *synonym* in ChEMBL, or a previously curated name in the *ct_nct_id2molregno* table, is manually curated as follows. All cases where the name (or synonym) matches a substring of the intervention name are identified (a “partial match”). Any name or synonym with less than four characters (typically an acronym) is excluded because these cases often map to multiple nondistinct intervention substrings. Similarly, trade names that commonly mismap to an intervention substring are excluded e.g., “astat” is a synonym of lanoconazole (CHEMBL1555126) but would be a substring match to “Atorv**astat** in 60 mg” (CHEMBL1487); “siran” is a trade name of acetylcysteine (CHEMBL600) but would be a substring match to “incli**siran**” (CHEMBL3990033). The identified partial matches are then manually examined and mapped to the appropriate drug or clinical candidate drug. In some cases, this step may require inspection of the full clinical trial information (e.g., NCT01251029), which usually includes the “Study Overview”, “Study Plan” and “More Information” sections.

For each clinical trial, the intervention is primarily mapped to a compound identifier (*molregno*). Alternatively, it could be mapped to (i) “-ABSENT-” which denotes that the compound is not currently available in ChEMBL, but may be included for the next ChEMBL release (if possible), or (ii) “-NOT-DRUG-” which denotes that the given intervention is not a drug. For example it may be a placebo (e.g., NCT02030977), control, surgery etc., or (iii) “-NOT-SPECIFIED-DRUG/S-” which denotes that the intervention_name does not name a specific drug, for example “Antibiotics”, “Antidepressants”, “Corticosteroids” (e.g., NCT01135641), etc. In cases where the intervention name does not clearly specify which drug or clinical candidate drug was administered, but other information available within the details of the trial does clearly specify the drug form, the intervention name may be flagged as equal to 1 in the *not_for_automatic_mapping* field of the *ct_nct_id2molregno* table so that the nonspecific intervention name is not reused in our dictionary for subsequent automated mapping. For complex clinical trials, where there are multiple drugs and multiple conditions being tested, an internal category system is manually assigned in the *is_indication* field of the *ct_nctid_condition_efo* table to directly map between an individual drug and a specified condition that is being studied.

The following paragraphs describe the detail of the condition mapping. For each clinical trial, the condition(s) that is being studied is mapped (by name) to an indication in EFO and stored in an internal *ct_nctid_condition_efo* mapping table that is not made publicly available. The *ct_nctid_condition_efo* table also acts as a curated dictionary of each condition and its equivalent EFO identifier. The version of EFO is updated for each ChEMBL release containing a drug update (using the EFO version described in the version table; EFO v3.56.0 was used for ChEMBL 34). In a similar manner to the intervention mapping, all exact matches of the condition against an EFO name or synonym are automatically mapped. However, any condition that does not exactly match an EFO name or synonym, or a previously curated condition in the *ct_nctid_condition_efo* table, are manually curated as follows. All cases where the EFO name or synonym matches a substring of the condition are identified (a “partial match”). Any name with less than four characters is excluded because these cases (typically acronyms) often map to multiple distinct conditions. Similarly, EFO terms that commonly mismap to a condition substring are excluded e.g., the EFO term “mental disorder” (EFO:0000677) would be an incorrect substring match to the condition “developmental disorder”, or the EFO term “small cell lung cancer” (EFO:0000702) would be an incorrect substring match to the condition “non small cell lung cancer”. The identified partial matches are then manually examined and mapped to the appropriate EFO identifier which may require inspection of the full clinical trial information for the curator to precisely understand the condition being studied.

In addition to the indication identifier (EFO id), the condition may be mapped to “-ABSENT-” which denotes that the indication is not currently available in the EFO, or “-NOT-CONDITION-” which denotes that the condition does not clearly specify an indication, but typically describes some other aspect of the trial, e.g., “colonoscopy” (e.g., NCT00164151), “vascular complications” (e.g., NCT05217654), “unresectable” (e.g., NCT01318642) etc. In cases where the condition does not clearly specify the indication, but other information available within the details of the trial does clearly specify the condition, the condition may be flagged as “*not_for_automatic_mapping*” so that nonspecific conditions are not added to our dictionary for use in subsequent automated mapping.

Clinical trials where only one condition is being studied can be straightforwardly assigned to one or more drugs. However, for individual clinical trials where there are multiple conditions and multiple drugs, a numerical category system is used to manually assign each drug to the condition for which it is being tested (in the *is_indication* field of the *ct_nctid_condition_efo* table). This step uses a combination of a script to determine similarity between conditions and manual inspection of the details of each clinical trial. If the trial has not been reviewed (for example, due to time constraints) then the multiple conditions and multiple drugs cannot be disentangled and the clinical trial will not be included in ChEMBL. The evaluation of semantic similarity is performed using the R package “ontologySimilarity”^64^ that uses the EFO obo file to calculate similarity between ontological terms. Based on expert judgment, if two conditions have a threshold similarity of 0.85 or higher, they are considered to accurately represent the condition being studied. For example, the clinical trial NCT00042848 lists two conditions: “Fatigue” and “Unspecified Adult Solid Tumor, Protocol Specific” that are being studied by the Phase 3 drug modafinil. The similarity of these conditions is less than 0.85 and therefore manual curation is required. For this trial, the first condition is manually inspected and flagged as *is_indication* = 1 (because the condition is considered to be correct and can be mapped to one drug) while the second is assigned *is_indication* = 0 to show that this is not the correct condition. Equally, the clinical trial NCT00005792 lists two conditions: “Multiple Myeloma” and “Plasma Cell Neoplasm” that are being studied for a combination therapy comprising three drugs. The conditions are deemed to be sufficiently similar (>0.85) so that both conditions are automatically assigned *is_indication* = 1 (because the conditions are each considered to be correct and can be mapped to one combined therapy comprising all three drugs).

The *redundant* flag is used to choose a more specific condition when multiple conditions are available, usually as ontological descendants of one another. For example, a phase 1 clinical trial (NCT01380756) tested the intervention AMG 900 (CHEMBL2140408) and lists four conditions: “Leukemia”, “Hematologic Malignancies”, “Myeloid Leukemia”, “Cancer”). In this case, three of the conditions are considered to be redundant (*redundant* = 1) because they are less specific, therefore only the condition “Myeloid Leukemia” (D007951/MONDO:0004643) is included in ChEMBL.

Note that clinical trial data are extracted as it is presented on ClinicalTrials.gov and, in some cases, this may lead to limitations where the clinical trial data are inaccurately reported. For example, the effect of dietary nitrate from beetroot juice is used as the intervention to study the condition of endothelial dysfunction that has been induced by typhoid vaccine (NCT02715635). However, the clinical trial inaccurately describes typhoid vaccine as the biological intervention and beetroot juice as a dietary supplement, rather than beetroot juice as the intervention.

Accurate curation of partially matched clinical trial intervention names and conditions is a time-consuming process. For example, ten months of new or updated clinical trials resulted in ∼ 8,000 trials, ∼ 25,000 partially matched intervention names, and ∼ 20,000 partially matched conditions that required manual inspection to provide accurate mapping. Our experience to date shows that there is no equivalent method using, for example natural language processing or text mining or large language models, that would provide a sufficiently accurate result. In addition, the process relies on high quality name and synonym curation in ChEMBL (and EFO) whereby any name or synonym is mapped to a single distinct drug or clinical candidate drug, or EFO indication. Therefore, the ongoing work to curate duplicate names or synonyms in ChEMBL is an important aspect of the Clinical Trials Pipeline (see Preferred Name, Synonym and Trade Name Curation).

## Incorporating the Clinical Trial Pipeline Data into ChEMBL and Open Targets

For each drug update of ChEMBL, the intervention names that have been mapped by the Clinical Trials Pipeline to a compound identifier (*molregno*) are loaded. Intervention names that are marked as “-ABSENT-” and cannot be found in ChEMBL are checked against GSRS to extract the chemical structure, if available. If a further structure check (using the standardized chemical structure^5^) against the existing chemical structures in ChEMBL yields no match, then the compound is registered as a new *molregno*. Any existing intervention that has been mapped to a recently downgraded compound identifier in ChEMBL is remapped to the current *molregno*. EFO is upgraded to the version required by the Open Targets Platform, and as a result, any conditions that have been mapped to an obsolete EFO identifier are remapped after manual inspection. Similarly, any EFO identifier that has been merged with another EFO identifier is also inspected and remapped to the correct EFO identifier. The primary classification used by ChEMBL to annotate indication data is Medical Subject Headings (MeSH^65^) and therefore all EFO terms are further mapped against equivalent MeSH terms.

For each clinical trial, the clinical development phase for the condition is loaded into the *drug_indication.max_phase_for_ind* field. The categories assigned for this field (and in *molecule_dictionary.max_phase*) are explained in more detail in the Indication and Maximum Phase of Development sections. However, it is worth noting that although regulatory bodies such as the FDA or EMA provide approval for a drug to treat a specified indication, the same drug may be studied in a clinical trial for a completely unrelated condition that has not been granted regulatory approval. For example, oxcarbazepine (CHEMBL1068) is approved by the FDA to treat seizures, but has also been studied in clinical trials to treat the distinct condition of bipolar depression. Therefore, ClinicalTrials.gov displays the clinical development phase as “Phase 4” (e.g., NCT03567681), although oxcarbazepine does not have regulatory approval as a marketed drug to treat bipolar depression.

The Clinical Trials Pipeline is run more frequently than the annual schedule of drug data updates that feed into ChEMBL. For the intervening iterations of the pipeline, clinical trial data are delivered directly to the Open Targets Platform as described below. The Open Targets Platform is a tool that supports systematic identification and prioritisation of potential therapeutic drug targets.^16^ All approved drugs or clinical candidate drugs that have an annotated mechanism and an indication in ChEMBL are delivered to Open Targets (regardless of their clinical development phase). The mechanism of action (e.g., inhibitor, agonist etc.) links a bioactive drug or clinical candidate drug to its therapeutic drug target. Within each compound family, the mechanism(s) and indication(s) data are aggregated upward. For example, if there is a mechanism and indication curated for a salt drug form, then the mechanism and indication are assigned to both the salt and the parent forms. However, if there is a mechanism and indication curated for the parent drug form only, then the mechanism and indication are only assigned to the parent form. For the Open Targets deliverable, the approved drug data are added to the clinical candidate drug data extracted from the Clinical Trials Pipeline. The approved drug sources of data include FDA Orange Book, FDA New Drug Approvals, EMA and ATC where an indication has been mapped in ChEMBL.

## Indication

An indication is the medical condition for which a medicine is used.^66^ It can include the treatment, prevention and diagnosis of a disease. The therapeutic or diagnostic indications for drugs and clinical candidate drugs in ChEMBL are mapped to equivalent terms in the Medical Subject Headings (MeSH^65^) controlled vocabulary. Note that off-label drug use is not curated as an indication, but may be captured as an aspect of the drug safety curation under the “misuse” category, see Drug Withdrawal and Warning Information.

MeSH is the primary identifier for an indication in ChEMBL and, along with EFO, has been used to map indications since release 21 when the *drug_indication* and *indication_refs* tables were introduced (Figure 4a). MeSH identifiers are publicly available for distribution and reuse (unlike e.g., MedDRA^67^ or SNOMED^68^) and are commonly used to describe approved drug indications within the drug discovery community. For example, EMA uses MeSH terms to describe the high-level therapeutic area for each medicinal product. In addition to MeSH, each indication is also mapped to the Experimental Factor Ontology (EFO) where possible. The use of EFO provides a common ontology tool that is used for many EMBL-EBI resources, and notably incorporates the Mondo Disease Ontology (Mondo^69^) that merges multiple resources to yield a coherent merged disease ontology.

**Figure 4:**
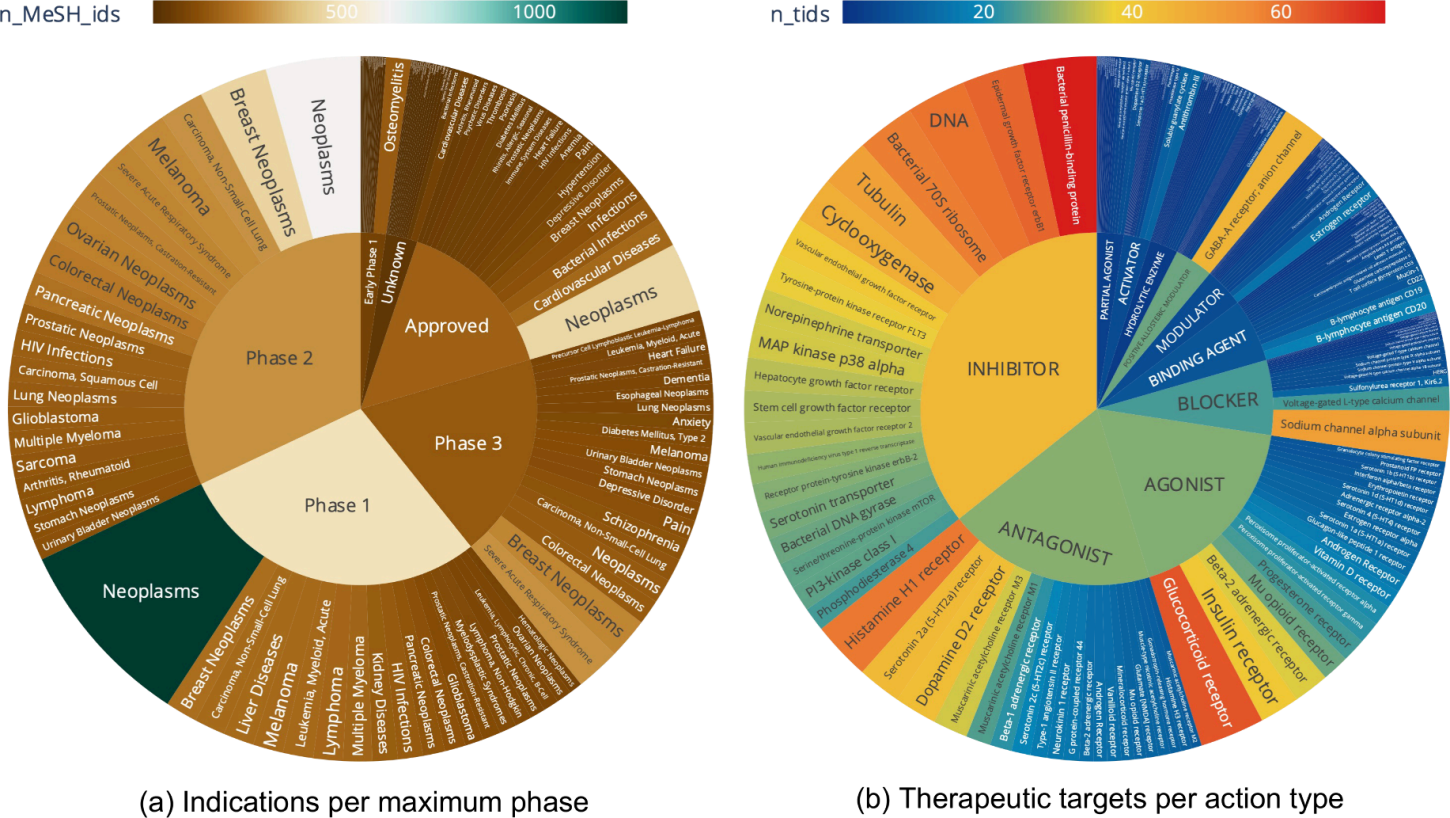
An overview of indications and therapeutic targets. (a) Counts of indications per maximum phase for drugs in ChEMBL 35. The plot shows the number of distinct MeSH identifiers for all drug forms in each compound family per maximum phase category. The inner labels are the categories of maximum phase. The outer labels are the MeSH headings that correspond to each MeSH identifier and the legend shows the number of distinct indications. The top 20 indications for each maximum phase category are shown. (b) Counts of therapeutic targets per action type for drugs in ChEMBL 35. The plot shows the number of distinct target identifiers (*tid*) for all drug forms in each compound family per action type. The inner labels are the categories of mechanism of action. The outer labels are the preferred target names that correspond to each target identifier and the legend shows the number of distinct targets. The top 20 targets per action type category are shown.

For each indication, the curation process typically involves reading the description given in the “Indications and Usage” or “Therapeutic indications” section of the medicinal product label for a specific drug form, or the “Therapeutic Claim” of a USAN or INN application, and manually mapping a word or phrase that describes the indication to an equivalent MeSH term (called a MeSH heading). The textual indication is only mapped to some branches of the MeSH tree, namely: C (Diseases), E (Analytical, Diagnostic and Therapeutic Techniques, and Equipment) or F (Psychiatry and Psychology), or selected specific terms like “Skin Aging” (under G13). If the description provides more than one disease term, then the most specific indication is mapped. For example, the USAN therapeutic claim for darovasertib is given as “Treatment of metastatic uveal melanoma and other tumors. . .” and would be mapped to “Melanoma” (D008545) in preference to “Neoplasms” (D009369). Note that it would not be mapped to “uveal melanoma” (C536494) because this is a MeSH supplementary concept. Prior to ChEMBL 32, disease indications were mapped (in the *drug_indication* table), while both disease and diagnostic interventions have been captured for more recent releases. The reference for each indication is captured in the *indication_refs* table which shows the original source of the information (e.g., FDA drug label, EMA medicinal product information, USAN application etc.). All MeSH and EFO identifiers are updated for each drug release of ChEMBL. This is a semiautomated process whereby a downgraded MeSH or EFO identifier is automatically remapped, but cases where two identifiers have been merged or their granularity changed by the ontology provider requires manual inspection and curation.

For each new FDA drug approval and any FDA drugs that have been recently added to the Orange Book, the phrase describing the therapeutic indication(s) is mapped to an equivalent MeSH term. For example, the medicinal product label for gepirone hydrochloride (CHEMBL1204187, 021164s000lbl) includes the phrase “is indicated for the treatment of major depressive disorder (MDD) in adults” and has been mapped to the MeSH heading: Depressive Disorder, Major (D003865). DailyMed^70^ is a database of labels for FDA drug products. DailyMed labels are typically reformatted to make them easier to read, and therefore indications for FDA approved drugs often show a reference in the *indication_refs* table with *ref_type* = “DailyMed”, or *ref_type* = “FDA” if a drug label is not available in the DailyMed database (for newly approved drugs, for example). The clinical development phase for each drug-indication pair is captured in the *max_phase_for_ind* field within the *drug_indication* table (see Maximum Phase of Development).

EMA drugs were first included in CHEMBL 34 and their indications were mapped using the MeSH term(s) that is provided as the “Therapeutic Area” by EMA. For example, EMA provides the MeSH headings: “Lymphoma, Non-Hodgkin” and “Hodgkin Disease” for brentuximab vedotin (CHEMBL1742994, adcetris) which were used to map the indications for ChEMBL. However, several inconsistencies in the EMA-assigned MeSH headings were noted and reported to EMA, and for future releases of CHEMBL the more detailed therapeutic description of the indication provided in the EMA Product Information will also be consulted.

ATC drugs are classified into five levels based on their anatomical, therapeutic and chemical properties. The level that contains the most detailed therapeutic description is used to extract the indication that is mapped to an equivalent MeSH term. Note that one drug may be assigned more than one ATC code, in which case multiple indications may be mapped. For example, cefamandole (CHEMBL1146) is described by ATC under level 2 as “antibacterials for systemic use” (J01DC03) and has been mapped to the MeSH heading “Bacterial Infections” (D001424).

When a USAN or an INN application is published, it may include a description of the therapeutic claim. For recent clinical candidate drugs, the text describing the therapeutic claim (if available) has been mapped to an equivalent MeSH identifier, and this source of indication information was first published for ChEMBL 34. For example, tazemetostat (CHEMBL3414621) has a USAN therapeutic claim that states “antineoplastic” and has been mapped to the MeSH indication for “neoplasms” (D009369).

For clinical candidate drugs that are undergoing clinical trials, the “condition” being studied in the Clinical Trial is extracted and mapped to an equivalent EFO identifier via the Clinical Trials Pipeline (see Mapping the Intervention Name(s) and Condition(s)). For integration into ChEMBL, each EFO identifier is also mapped to an equivalent MeSH heading. In some cases, an equivalent MeSH term cannot be mapped due to a mismatch between the levels of granularity in MeSH versus EFO, and a curation decision may be taken to map to a higher, less-specific, MeSH term. In some cases, it may not be possible to map to an equivalent MeSH term, which would mean that a drug-indication pair for the clinical trial would not be incorporated into ChEMBL. For example, the clinical trial NCT02543099 uses policosanol (CHEMBL3707352) to study endothelial dysfunction (EFO:1001461), but no equivalent MeSH heading can be mapped and therefore the trial is not included in ChEMBL.

Our current process to curate indications is a manual, resource-intensive process that cannot be easily updated across multiple versions of medicinal product labels that are generated over time e.g., acetaminophen has ∼ 5000 FDA medicinal product labels currently. For example, later iterations of each drug label may include additional, distinct indications that should be also captured, or a drug may subsequently be repurposed to treat an entirely different indication. Ideally, a stepwise process of an initial step of automated text mining to capture an appropriate indication and suggest an ontology term, followed by manual inspection and definitive term mapping by one (or more) human curators for tricky cases would maintain the high-quality, accurate result that we require. For ChEMBL 30, some indications were automatically assigned using an NLP approach to extract indications from DailyMed medicinal product labels, but this was subsequently noted to have inconsistencies in the drug form being annotated, and the indication being assigned, and the method was not considered sufficiently robust for regular production code. Given the fast-moving evolution of text mining tools for biomedical entities and mapping to an ontology (e.g. refs 71 and 72), ChEMBL is continuing to work toward methods to improve the efficiency of its indication curation.

## Mechanism of Action

A mechanism of action is assigned to link a drug or clinical candidate drug to its therapeutic target(s) which is the target responsible for the effect on a disease. Data are captured in the *drug_mechanism* and *mechanism_refs* tables (Figure 4b). The mechanism of action is manually curated, and one drug may have more than one therapeutic target, each with a distinct mechanism of action. All targets listed in the mechanism of action section of a reference, such as an FDA drug label, would be captured. Antibody drug conjugates or bispecific antibodies are likely to have multiple targets. For example, cantuzumab mertansine (CHEMBL1742997) is an inhibitor of tubulin and a binding agent of Mucin-1. For drug mechanisms that are unknown, nonmolecular, or have not been fully elucidated, a summary is included in the *mechanism_of_action* field (e.g., Unknown, Antioxidant, Diagnostic, Laxative) with any specific details included in the *mechanism_comment* field. For example, tozuleristide is an imaging agent and has been given a “diagnostic” *mechanism_of_action* (CHEMBL3990023). In future, the *mechanism_comment* will also give further information about a target-indication pair where one target is implicated for one indication and a different target for a different indication for the same compound. As mentioned above (see Indication), later iterations of a drug label may include additional, distinct indications but these are not always captured using our current process. A goal for future pipelines is to capture the mechanism(s) for any additional indication(s). For example, imatinib mesylate (CHEMBL1642) has 70 indications in ChEMBL 35, including a single indication at max_phase 4 (chronic myelogenous leukemia) for which the drug was originally approved. However, a recent drug label reveals that the drug is now approved for several other indications including Kit+ Gastrointestinal Stromal Tumors. The primary target is most likely BCR-ABL for the initial approval against Philadelphia Positive Chronic Myeloid Leukemia but KIT for the Kit+ Gastrointestinal Stromal Tumors indication.

The target identifier is specified in the *tid* field (foreign key to the *target_dictionary* table), but if the drug has a nonmolecular therapeutic target (i.e., antioxidant, supplement, diagnostic), then the *tid* is left as NULL. If known, the binding site for the drug within the target is specified in the *site_id* field (foreign key to *binding_sites* table). This is usually applicable to protein complex targets (e.g., Interleukin-2 receptor is the protein complex target of the drug aldesleukin, CHEMBL1201438), with additional information provided in the *binding_site_comment* field. The type of action of the drug on the target (e.g., agonist, antagonist, degrader, sequestering agent, vaccine antigen, etc.) is provided in the *action_type* field (foreign key to the *action_type* table). For ChEMBL 34, two new *action_type* categories were included to describe gene and protein therapies, namely exogenous gene and exogenous protein.

Comments about the selectivity of the drug against the target (e.g., target enzyme selectivity, variant selectivity or target organism selectivity) are included in the *selectivity_comments* field. If a drug is selective against a variant target over the wild-type, then the *variant_id* is specified in the *drug_mechanism* table (foreign key to the *variant_sequences* table). For example, if the drug target is epidermal growth factor receptor (EGFR) with a resistance mutation (T790M) then this information is captured in the *variant_id* field, such as for firmonertinib, CHEMBL4297258). For an undefined variant target such as a deletion mutant, the *variant_id* is recorded as −1 (an undefined mutation).

Typically, a medicinal product label is the reference source for approved drugs, while clinical candidate drugs may cite a scientific journal article. Drug mechanisms that were curated during the early years of ChEMBL may cite a company Web site or similar, and our curation team is working to update these references where possible.

## Drug Withdrawal and Warning Information

Drug safety data continues to be curated for each ChEMBL release and is captured in the *drug_warning* and *warning_refs* tables (Figure 5). For convenience, a flag is also available in the *molecule_dictionary* table, called *withdrawn_flag* or *black_box_warning*.

**Figure 5:**
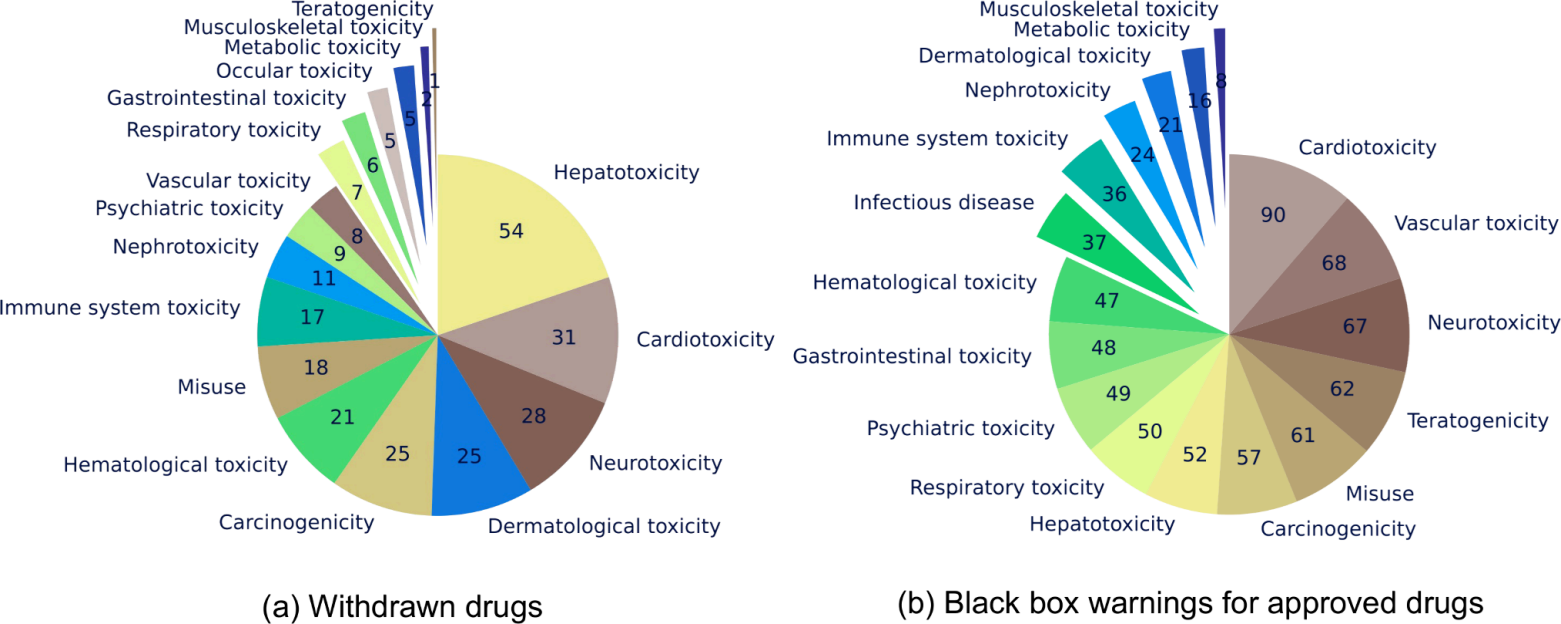
Drug warning data for CHEMBL 35. (a) The number of withdrawn drugs for each toxicity category. The labels show the count of distinct parent drugs. (b) The number of approved drugs that carry a black box warning for a severe or life-threatening adverse effect for each toxicity category. The labels show the count of distinct parent drugs.

The set of approved drugs that have subsequently been withdrawn from the market for safety reasons (“withdrawn drugs”) was fully reviewed for ChEMBL 32, and our rules were updated, clarified and formally written, as described below.

In ChEMBL, a withdrawn drug is an approved drug that has subsequently been withdrawn for toxicity reasons. Only human drugs are curated for their withdrawn status, and not veterinary drugs, for example. A drug is assigned as “withdrawn” if

- All doses are withdrawn (and not just the highest dose).
- The drug is withdrawn for all populations (and not just infants).
- The drug is withdrawn for all indications.
- Any drug withdrawn for a lack of evidence of efficacy is not included.
- Any drug withdrawn for drug–drug interactions is included if it is a safety-related withdrawal.

A regulatory agency (e.g., EMA, FDA) is the preferred source of information for the withdrawn status, but some worldwide sources of information are consulted from e.g., WHO. The withdrawn status is mapped to an individual drug form (e.g., a parent or salt drug form). Terminology such as “banned”, “prohibited” or “revoked” is considered to be equivalent to withdrawn status, however, the mention of “suspended” requires additional source references or checks to confirm that the drug was not reintroduced. The earliest year of withdrawal is assigned as the *warning_year*. The region of the world that the withdrawn status applies to is recorded as the *warning_country*.

Each drug flagged as withdrawn includes a citation to a regulatory document or similar. The specific (granular) reason for withdrawal is mapped to EFO, and a high-level toxicity class is also assigned. For example, for tergenadine (CHEMBL17157), the phrase “cardiac arrthymia” would be mapped to EFO (EFO:0004269), and “cardiotoxicity” would be assigned as the high level *warning_class*, and mapped to EFO (e.g., EFO:1001482).

There are 202 parent drugs flagged as withdrawn for ChEMBL 35. For example, nefazodone hydrochloride (CHEMBL1200492) was withdrawn in 2003 from Canada for causing adverse hepatic events. However, it remains available in the United States but carries a black box warning for hepatoxicity and psychiatric toxicity. 38 drugs were newly flagged as withdrawn using the revised rules, for example, almitrine (CHEMBL1183717) or benzbromarone (CHEMBL388590). In contrast, 12 drugs that were previously flagged as withdrawn did not meet the updated selection criteria and therefore were no longer considered withdrawn (e.g., CELECOXIB (CHEMBL118) which has not been withdrawn for all indications, or diethylstilbestrol (CHEMBL411) which is still used to treat prostate cancer).

In addition to the withdrawal status of drugs, ChEMBL also assigns black box warning data from FDA drug labeling documents. FDA drugs carry a black box warning if they have a severe or life-threatening side effect(s). The warnings are assigned a high level toxicity class using natural language processing (NLP) models.^73^ For example rosiglitazone maleate (CHEMBL843) carries a black box warning for cardiotoxicity in the United States, but has been withdrawn from other regions of the world, including the European Union, for increased risk of ischemic heart disease. For ChEMBL 32, the high level warning class for each black box warning was mapped to EFO, and there were some minor changes of warning class names as a result of expert toxicological review: gastrotoxicity is now called gastrointestinal toxicity (EFO:0011050), metabolism toxicity is now called metabolic toxicity (EFO:0011054), and misuse is now called drug misuse (EFO:0011049).

The NLP models are currently in the process of being updated to the latest model architectures available in spaCy,^74,75^ and Python 3 environment. The manually curated training set, previously compiled using data up to 2020, is being expanded with drug labels published more recently and novel drug approvals since 2020. The new predictions are planned to be published in ChEMBL 36.

## Drug Property Information

For each compound in ChEMBL, a set of compound-related properties is available in the *molecule_dictionary* table. Many of these properties are primarily assigned for drugs or clinical candidate drugs, and this section describes these drug properties and the underlying curation methods. In the internal “Drugbase” database, every drug property is assigned to an individual drug form (e.g., a salt). However, when the drug property data are integrated into the overall ChEMBL database, all individual drug form data become aggregated onto the parent drug form (except for Dosed Ingredient). This approach means that any virtual parent compound has drug property information available in ChEMBL which would not otherwise be the case. Equally, many aspects of drug discovery tend to group drug attributes onto the parent drug since the bioactivity will remain similar regardless of the counterion of each individual drug form.

## Maximum Phase of Development

In ChEMBL, *max_phase* is defined as the maximum phase of development reached for the compound across all indications (in the *molecule_dictionary* table, Figure 6). For example, mitapivat sulfate (CHEMBL4297223) is an FDA and EMA drug that is approved to treat hemolytic anemia, congenital hemolytic anemia and inborn genetic diseases. Therefore, *max_phase* category 4 is assigned to mitapivat sulfate. The parent, mitapivat (CHEMBL4299940) has been studied in Phase 1 clinical trials onward for alpha- and beta-thalassemia, anemia, thalassemia, sickle cell anemia and liver disease, but is not approved by a regulatory agency for any of these indications. However, the parent, mitapivat, is also assigned max_phase category 4 because property data for individual drug forms are aggregated onto the parent drug.

**Figure 6:**
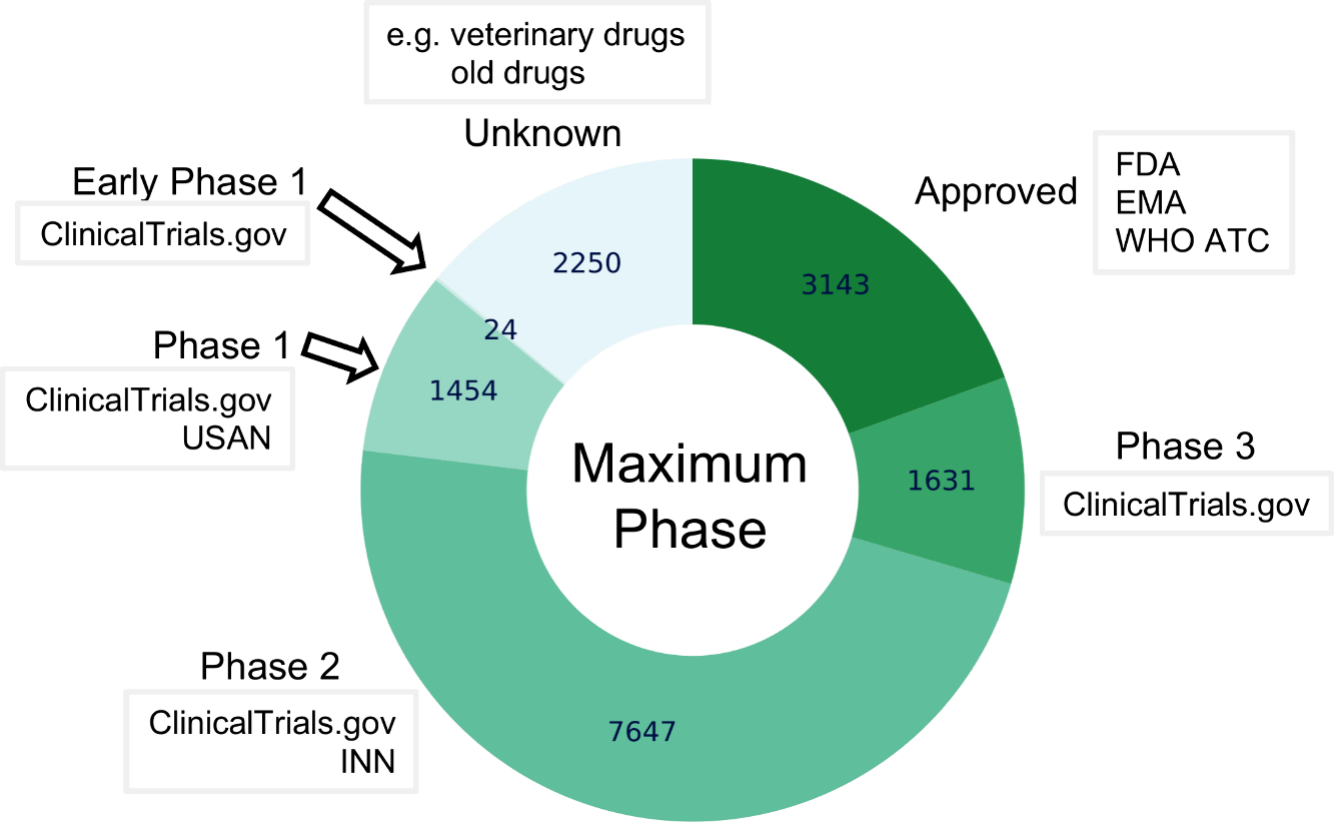
Number of distinct parent drugs assigned to each maximum phase category and the source of this data.

Our rules to assign the *max_phase* categories have recently been reviewed, clarified and formally written (see below), and will be applied to new drugs or to any manually curated updates for existing drugs.

*Max_phase* category 4.0 is assigned for drugs that have been approved by a regulatory agency for a specified indication. This includes all FDA drugs (e.g, FDA Orange Book, FDA New Drug Approvals and FDA Biological License Approvals, and FDA nonprescription drugs such as those approved via the Over-The-Counter drug monographs^76^), all EMA medicinal products that have been approved for human use (i.e., not those EMA drugs that are only approved for veterinary use), and Withdrawn drugs (because for a drug to be curated as “withdrawn” in ChEMBL, it must have been approved in at least one region of the world). Other regulatory agencies such as the Japanese PMDA,^77^ or UK MHRA^78^ may also be used to assign *max_phase* 4.0 for approved drugs even if these sources of drug information are not routinely incorporated into ChEMBL at present. The WHO is not a regulatory agency for drug approval, but it maintains the ATC list of drugs which provides an “international gold standard for drug utilization studies in an effort to ensure universal access to essential drugs”,^79^ i.e., a list of essential drugs used worldwide. ATC drugs are usually described as the parent drug form. Therefore, if the drug is an ATC and there is no other regulatory source described in ChEMBL within the compound family, then *max_phase* category 4 is assigned.

*Max_phase* category 3.0 is assigned for clinical candidate drugs in Phase 3 clinical trials that are extracted via the Clinical Trials Pipeline.

*Max_phase* category 2.0 is assigned for (i) clinical candidate drugs in Phase 2 clinical trials (or in Phase 2/Phase 3 clinical trials) that are extracted via the Clinical Trials Pipeline, and (ii) INN applications because their guidance that states “As a general guide, the development of a drug should progress up to the point of clinical trials (phase II) before an application is submitted to the INN Secretariat for name selection.”.^45^

*Max_phase* category 1.0 is assigned for (i) clinical candidate drugs in Phase 1 clinical trials (or in Phase 1/Phase 2 clinical trials) that are extracted via the Clinical Trials Pipeline, and (ii) USAN applications because their guidance states “Firms usually apply for a USAN when the investigational therapy is in Phase I or Phase II trials”.^41^

*Max_phase* category 0.5 is assigned for clinical candidate drugs in Early Phase 1 clinical trials that are extracted via the Clinical Trials Pipeline.

*Max_phase* category −1 (unknown) is assigned where ChEMBL cannot assign a development phase. For example, unknown status could be assigned if the compound is not regulated for human medicine (e.g., a veterinary drug or an environmental chemical) or is an old compound that progressed through clinical trials prior to the first release of the ClinicalTrials.gov resource. Research compounds with bioactivity data are assigned NULL in the *max_phase* field (i.e., these are not drugs or clinical candidate drugs).

The *max_phase_for_ind* is defined as the maximum phase of development that the drug is known to have reached for this particular indication. It is available in the *drug_indication* table. The *max_phase_for_ind* categories are the same as those given above for max_phase.

Although regulatory bodies such as the FDA or EMA provide approval for a drug to treat a specified indication (assigned *max_phase_for_ind* 4.0 in ChEMBL), the same drug may be studied in a clinical trial for a completely unrelated condition that has not been granted regulatory approval. For example, oxcarbazepine (CHEMBL1068) is approved by the FDA to treat seizures, but has also been studied in clinical trials to treat the distinct condition of bipolar depression (e.g., NCT03567681). In Clinical Trials, Phase 4 is defined as “a phase of research to describe clinical trials occurring after FDA has approved a drug for marketing.”^80^ Therefore, ClinicalTrials.gov displays the clinical development phase as “Phase 4”, although oxcarbazepine does not have regulatory approval as a marketed drug to treat bipolar depression. To date, ChEMBL has excluded all indications for a drug in Phase 4 clinical trials unless the indication has been approved by a regulatory agency.

## Dosed Ingredient

*Dosed_ingredient* (in the *molecule_dictionary* table) is a flag to indicate that the drug is administered to the patient in this form (e.g., the salt drug form, where 1 = yes or 0 is the default value). This flag only applies to approved drugs for human use from regulatory agencies like the FDA or EMA and not to veterinary drugs or agrochemicals for example. Unlike other drug property data, the *dosed_ingredient* flag is not aggregated onto the parent drug form. Therefore, the *dosed_ingredient* data can be used to distinguish whether an approved parent drug is administered to the patient, or not.

## Molecule Type

The drug discovery community broadly distinguishes between a small molecule compound and a biotherapeutic compound (e.g.,^81,82^ ). In ChEMBL, a small molecule is loosely defined as any bioactive compound (usually an organic molecule that is chemically synthesized) where the chemical structure can be precisely drawn, and it is not a biotherapeutic with a complex structure that is not easily identified or characterized. The use of a molecular weight threshold (such as 900 Da) to distinguish between a small molecule and a biotherapeutic is not practical because a “small” molecule can have a large molecular weight. For example, peplomycin sulfate (CHEMBL3989633) has a clearly defined chemical structure given in the USAN application from 1981 with a high molecular weight of 1572 Da, or cefiderocol sulfate tosylate (CHEMBL4297211) has a clearly defined chemical structure given in the USAN application from 2017 and a very high molecular weight of 3062 Da.

Based on the inherent difficulties of an imprecise definition of a small molecule, ChEMBL instead applies a practical approach to determine the *molecule_type* category for drugs and clinical candidate drugs (in the *molecule_dictionary* table). The *molecule_type* category that is assigned can be one of: small molecule, inorganic small molecule or polymeric small molecule, antibody, antibody drug conjugate, cell-based, enzyme, gene, oligosaccharide, oligonucleotide, protein, or unknown (Figure 7a, Figure 9b).

**Figure 7:**
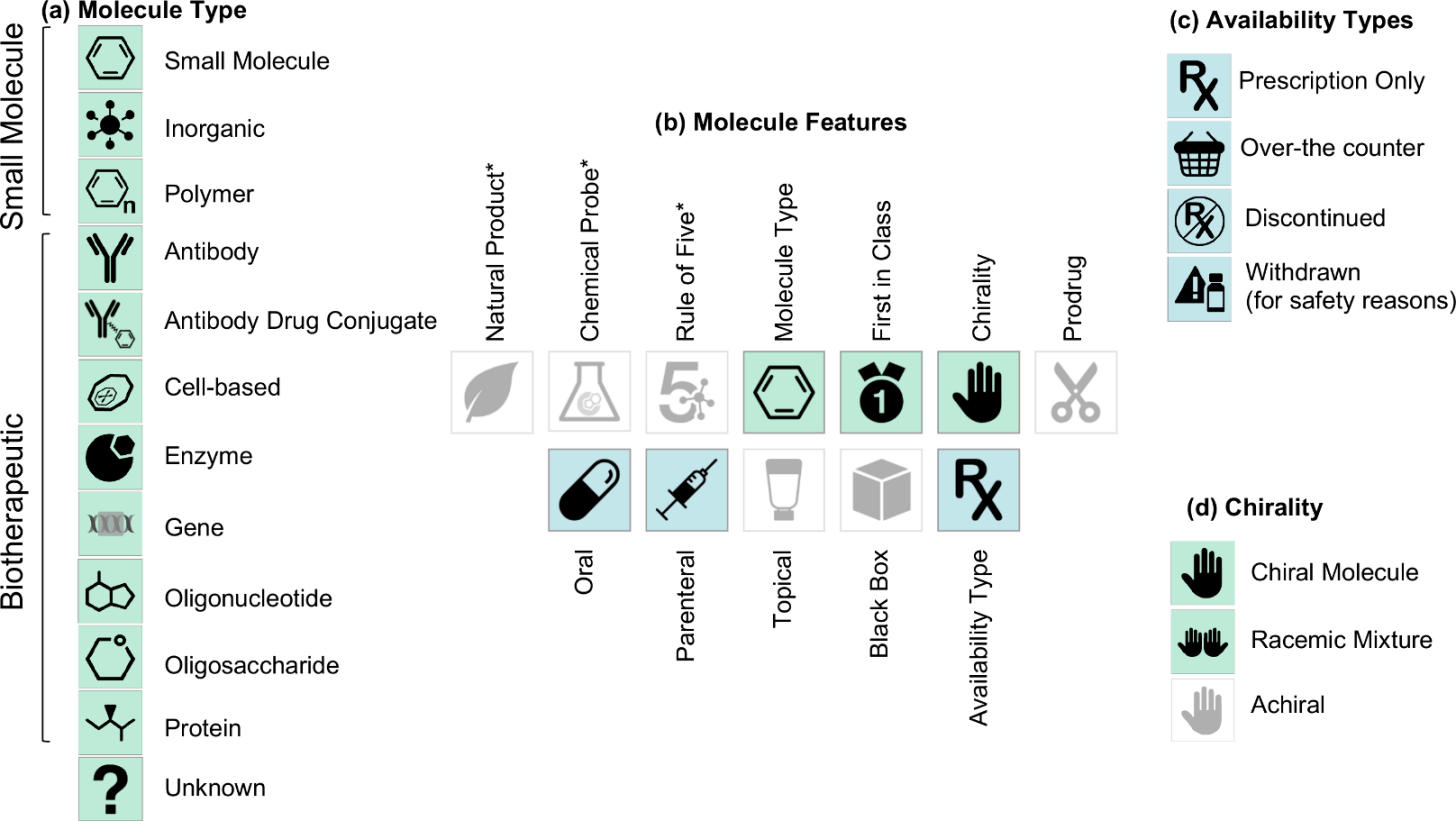
Drug properties and other molecule features. (a) The molecule_type categories. (b) An example of the molecule features shown on the compound report card for the approved drug, lenacapavir sodium (CHEMBL4802249). Each molecule icon is depicted as a yes (colored background) or no (uncolored background) unless it is for *molecule_type*, *availability_type* or *chirality* as shown in (a), (c) and (d). *Note that all compounds in ChEMBL, regardless of whether it is a drug or clinical candidate drug, or not, are assigned the natural product flag, chemical probe flag and the flag to show rule-of-five compliance for a drug-like molecule. (c) The *availability_type* categories. (d) The *chirality* categories.

Biotherapeutic compounds in ChEMBL usually do not have a chemical structure and therefore the *molecule_type* is typically based on the drug or clinical candidate name. For example, vofatamab is an antibody based on the “-mab” suffix (CHEMBL4297848), or ranpirnase is an enzyme based on the -ase suffix (CHEMBL2108479). For any biotherapeutic compounds that have a chemical structure, the *molecule_type* is based on (i) a chemical substructure match or (ii) the drug or clinical candidate name, followed by manual curation to assign only one *molecule_type* if these do not result in the same category. For example, fondaparinux sodium (CHEMBL1200644) is assigned as an oligosaccharide based on a chemical substructure search (using SMARTS), and agatolimod sodium (CHEMBL2103793) is assigned as an oligonucleotide based on a chemical substructure search (using SMILES). For cases where the biotherapeutic *molecule_type* is unclear, GSRS is consulted, and if required, may require further discussion by the curation team.

For the remaining drugs and clinical candidate drugs, the category “small molecule” is assigned if the compound has a chemical structure in ChEMBL and has not already been assigned as a biotherapeutic compound (see above). In addition, there are two further subclasses of “small molecule” that are shown in the *polymer_flag* and *inorganic_flag* fields of the *molecule_dictionary* table. The *polymer_flag* is assigned by a combination of chemical substructure search or the drug or clinical candidate name. The *inorganic_flag* is assigned when the chemical structure does not contain any atoms that are commonly found in organic compounds, namely H, C, N, O, P, S, F, Cl, Br, I. Any remaining compounds where the *molecule_type* cannot be clearly identified are assigned as “unknown”.

Regardless of the *molecule_type* category, manual inspection and if required, further curation is carried out in addition to the automated process. The manual curation oversight of molecule_type led to the introduction of a new category for ChEMBL 34 (“antibody drug conjugate”). As the modalities of drugs and clinical candidate drugs become more diverse (Figure 9b), there is likely to be a need to introduce further *molecule_type* categories. Currently, the *molecule_type* is routinely curated for drugs and clinical candidate drugs via our internal “Drugbase” database. By contrast, research compounds with bioactivity data in ChEMBL are not assigned a *molecule_type*, with the exception of nearly 21,000 research compounds that were assigned as “protein” based on their HELM monomer notation for peptide subunits (last updated for ChEMBL 25). The inconsistency in curation between *molecule_type* for research compounds versus drugs and clinical candidate drugs remains an area for future improvement, as resources allow.

## First in Class

In ChEMBL, *first_in_class* shows the first approved drug for a particular mechanism of action that links the drug to a therapeutic target, regardless of the indication, the route of administration or the type of molecule (in the *molecule_dictionary* table, where 1 = yes, 0 = no and −1 = research compound, i.e., not a drug, Figure 7). The *first-in-class* flag was first introduced in ChEMBL 15. *First_in_class* is manually curated for approved drugs only. Currently, the FDA summary reports on new drug approvals are the main source of *first-in-class* information (e.g.,^83^). There are 294 drugs annotated with first-in-class for ChEMBL 35, corresponding to 247 parent drug forms.

The rules for assignment of *first-in-class* are currently under review and, when clarified and formally written, will be applied to new drugs or to any manually curated updates for existing drugs. *First-in-class* curation for existing drugs has typically used the following rules but these may not have been consistently applied: (i) An approved drug with a new route of administration is not considered to be *first-in-class*. For example, nusinersen sodium (CHEMBL3833342) was the first SMN2 pre-mRNA positive modulator to be approved (in 2016 by FDA and EMA) for spinal muscular atrophy. It is administered parenterally and is flagged as *first-in-class* by ChEMBL. By contrast, risdiplam (CHEMBL4297528) was later approved for spinal muscular dystrophy by FDA and EMA (in 2020) against the same target. It is administered orally and is not considered to be *first-in-class*. (ii) No distinction is made for a target with a new *molecule_type*. For example, the *first-in-class* antibody, erenumab (CHEMBL3833329), was approved for migraine disorders in 2018 and is an antagonist for the calcitonin gene-related peptide type 1 receptor. In 2019, the small molecule, ubrogepant (CHEMBL2364638), was also approved for migraine disorders on the same target but is not considered to be *first-in-class*. (iii) In the scenario with a newly approved drug with multiple targets, and one of these targets is novel, then the drug would be considered as to be first-in-class. (iv) A diagnostic drug is not usually used to treat disease and therefore would not normally be considered as *first-in-class*. For example, gadopentetate dimeglumine (CHEMBL1200431) is commonly used as an imaging agent in magnetic resonance imaging. It is not flagged as a *first-in-class* drug despite being introduced as the first FDA MRI contrast agent in 1988,^84^ and patented in 1981 (US-5021236-A). (v) Finally, *first-in-class* is usually assigned only if the drug has been extracted from one of the sources of approved drug data in ChEMBL. For example, amenamevir (CHEMBL4297592) is a PMDA drug approved in 2017 for treatment of Herpes zoster and inhibits the herpes simplex virus (HSV)-1 helicase–primase complex,^85^ but is not currently flagged as *first-in-class* in ChEMBL.

## Chirality

There are different types of isomers. Stereoisomers and conformational isomers have the same molecular formula and the same connectivity between atoms but differ in the spatial arrangement of their atoms. Conformational isomers differ by rotation around single bonds while stereoisomers are interconverted by breaking or reforming bonds. Stereoisomers are described by the concept of *chirality* (in the *molecule_dictionary* table, Figure 7d), which is curated for drugs and clinical candidate drugs. A molecule may have multiple chiral atoms as follows:

- an achiral molecule (*chirality* = 2). There are either no chiral centers in the molecule, or the molecule contains chiral atoms and an internal mirror plane of symmetry giving an overall achiral molecule. For example, phetharbital (CHEMBL93218) does not contain a chiral center; it has two identical ethyl substituents on the diazine ring, adinazolam (CHEMBL328250) contains no chiral centers, or varenicline (CHEMBL1076903) contains two chiral centers but has an internal mirror plane of symmetry giving an overall achiral molecule.
- a single stereoisomer (*chirality* = 1). All stereocenters within the compound have known absolute configuration (e.g., levosulpiride, CHEMBL267044 the l-enantiomer with one stereochemical center, or moxifloxacin hydrochloride CHEMBL1200735 has two stereochemical centers with known absolute configuration). Most biological compounds are chiral. For example, nucleotides and ribonucleotides usually have R-configuration while amino acids and proteins usually have S-configuration. As a result, biotherapeutic compounds that do not have a chemical structure in ChEMBL are normally assigned as a single stereoisomer. For example, rasburicase, an enzyme and protein, CHEMBL1201594, and abaloparatide, an oligopeptide CHEMBL3301581.
- a mixture of stereoisomers (*chirality* = 0). This can include: (i) a racemic mixture where there is a 1:1 ratio of enantiomers (mirror image compounds and not superimposable), e.g., bevantolol hydrochloride (CHEMBL2106060), or (ii) a mixture of diastereoisomers (not mirror image compounds and not superimposable), e.g., zenidolol (CHEMBL513389), or (iii) a single enantiomer where all stereocenters have known relative configuration (i.e., not known absolute configuration), e.g., azilsartan mepixetil (CHEMBL5095053), etc., or
- has unknown chirality (*chirality* = −1). This category contains compounds where the chirality of the chemical structure is unknown or has not (yet) been assigned in ChEMBL.

C*hirality* is assigned by a combination of manual curation (for new USAN and INN compounds) and semiautomated assignment followed by manual checking and correction for other drugs and clinical candidate drugs. For example, if the InChIKey includes the string “-UHFFFAOYSA-” then the compound does not contain stereochemistry and could be assigned as either achiral or a mixture of stereoisomers (remember that for compounds where the proportions of stereoisomers are not precisely known, ChEMBL removes all stereochemical representation from the chemical structure). The manual curation process includes inspection of the chemical structure, and inspection of the systematic chemical name that may be described as RS, SR or ± for racemates or R/S, (±) for nonracemates, or D,L,DL,D/L etc. Tricky cases are discussed within the curation team, and GSRS may be consulted as an additional source of information. Within each compound family, the assigned chirality is usually identical for all drug forms.

## Prodrugs and Drug Metabolism Data

A drug that is metabolized into its pharmacologically active ingredient is termed a prodrug and shown in the *prodrug* field (in the *molecule_dictionary* table, where 1 = yes, 0 = no and −1 = research compound, i.e., not a drug, Figure 7). Prodrugs may include: (i) small molecules such as esters have improved lipophilicity and are hydrolyzed *in vivo* to release the active metabolite (e.g., mycophenolate mofetil CHEMBL1456 ^86^), (ii) ProTides allow delivery of nucleosides by masking the monophosphate/monophosphonate groups until delivery into cells (e.g., tenofovir alafenamide CHEMBL2107825 ^87^). Protoxins require enzymatic cleavage to generate the active toxin (e.g., topsalysin CHEMBL2364656,^88^). Some prodrugs are activated in specific microenvironments, such as the hypoxic tumor environment (e.g., evofosamide CHEMBL260046,^89^ or are activated by tumor associated proteases to reduce toxicity at nontarget sites in the patient (e.g., topsalysin CHEMBL2364656 ^88^).

As well as the *prodrug* flag, the pharmacologically active ingredient of a prodrug (source id = 53) is recorded as the *active_molregno* (in the *molecule_hierarchy* table, for ChEMBL 28 onward). For example, the approved drug nabumetone (CHEMBL1070) is a prodrug that is metabolized within the human body,^90^ to its pharmacologically active metabolite, 6-methoxy-2-naphthylacetic acid (CHEMBL1105, Figure 8a). This relationship can be accessed via the “Prodrug” section of the compound report card on the web interface (CHEMBL1070), or via the *molecule_hierarchy* table where nabumetone is shown in the *molregno* field and 6-methoxy-2-naphthylacetic acid is shown in the *active_molregno* field. The current database schema only allows for annotation of a single pharmacologically active ingredient for each prodrug in the *molecule_hierarchy* table, so intermediate pharmacologically active ingredients, or cases with multiple pharmacologically active end ingredients, are not stored. For example only the mertansine (CHEMBL4802230) drug component of the cantuzumab mertansine antibody-drug conjugate (CHEMBL1742997) is stored, and not the antibody component (cantuzumab). References for each prodrug and its pharmacologically active ingredient are stored internally and are available on request; these are typically either a medicinal product label or a scientific article.

**Figure 8:**
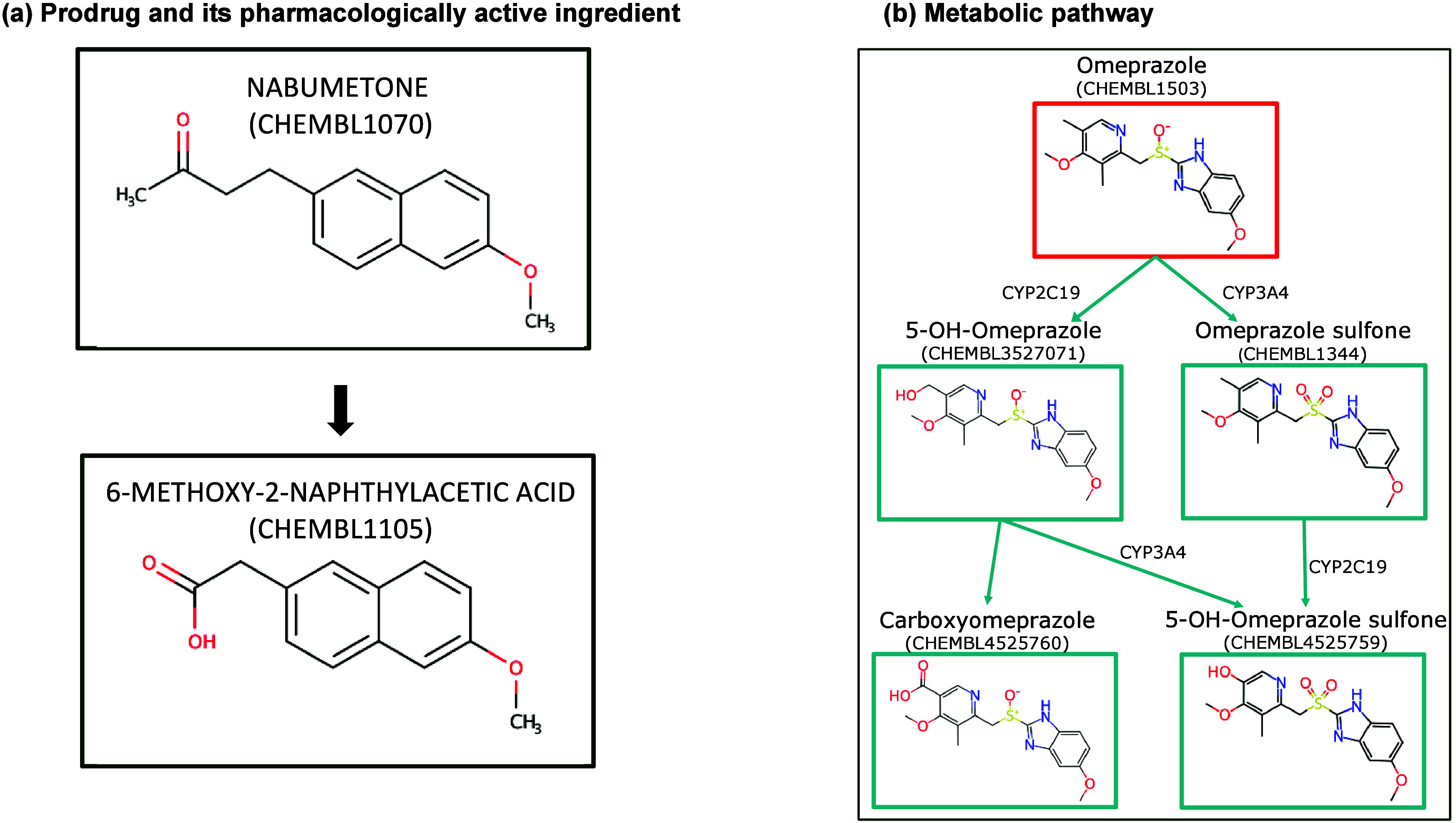
(a) Example of a prodrug nabumetone (CHEMBL1070) and its pharmacologically active ingredient (6-methoxy-2-naphthylacetic acid, CHEMBL1105). (b) Example of the approved drug omeprazole (CHEMBL1503) and its curated metabolic pathway showing its metabolites (including intermediate metabolites).

For any prodrug that is a member of a compound family with more than one drug form, all forms of the drug are assigned as a prodrug because they will metabolize within the human body to produce the same pharmacologically active ingredient. For example, tenofovir disoproxil fumarate (CHEMBL1486) is a prodrug that is metabolized within the human body to tenofovir diphosphate (CHEMBL258501), as shown in the FDA drug label (e122435e-cd0b-4c90–940a-b7a0d090d866). There are two further drug forms within the compound family: the parent tenofovir disoproxil (CHEMBL1538) and the salt tenofovir disoproxil aspartate (CHEMBL4594453); all three drug forms are assigned the *prodrug* = 1 flag. Note that there is one further (research) member of the compound family (CHEMBL3185360) but this is not currently assigned as a prodrug because the curation is only performed for drugs or clinical candidate drugs. There are 422 prodrugs annotated for ChEMBL 35, corresponding to 320 parent drug forms.

Metabolic pathway data are available in ChEMBL for a limited number of drugs (in the *metabolism* and *metabolism_refs* tables). Currently, there are 257 drugs (229 parent drugs) available with metabolic pathway data, however these data are not routinely updated and ChEMBL 28 included the most recent major curation of metabolism data. The data are extracted from the scientific literature (e.g., Journal of Drug Metabolism and Disposition), or from drug labels. For example, omeprazole (CHEMBL1503) is metabolized by two CYP enzymes to produce two intermediate metabolites that are broken down into two further metabolite products, Figure 8b. An interactive metabolic pathway diagram can be accessed via the “Metabolism” section of the relevant compound report card on the web interface.

## Route of Administration

The route of administration of an approved drug is shown in the *oral*, *parenteral* and *topical* fields (in the *molecule_dictionary* table, where 1 = yes and 0 is the default value for each field, Figure 7). For example, isosorbide dinitrate (CHEMBL6622) is available as an oral (by mouth) or topical (via the skin or mucous membrane) prescription drug, but is not available parenterally (via an intravenous, intramuscular, intrathecal or subcutaneous route). These data are mainly curated for FDA Orange Book drugs, with some additional manual curation arising from project-specific work e.g.,.^5^

## Availability Type

The marketing status of an approved drug is shown in the *availability_type* field (in the *molecule_dictionary* table, Figure 7c). The *availability_type* may be shown as

- 2 = An over-the-counter drug. For example acetaminophen (CHEMBL112) is available as an over-the-counter drug (e.g.,^91^). This category is currently curated for FDA Orange Book drugs only.
- 1 = A prescription drug. For example, oxaprozin (CHEMBL1071) is indicated for arthritis and is available as a prescription drug (e.g.,^92^). This category is currently curated for FDA Orange Book drugs only.
- 0 = A drug that has been discontinued. For example phenmetrazine hydrochloride (CHEMBL1200483, e.g.,^93^) is an approved drug product that shows a discontinued marketing status. This category is currently curated for FDA Orange Book drugs only. Note that FDA describes a “discontinued drug product” as approved products that have never been marketed, have been discontinued from marketing, are for military use, are for export only, or have had their approvals withdrawn for reasons other than safety or efficacy after being discontinued from marketing.^94^
-1 = The marketing status of the drug is unknown. This category may be assigned if it is a clinical candidate drug that has not been approved, or is an approved drug from a non-FDA Orange Book source of information. For example, the EMA drug, parecoxib sodium (CHEMBL296913) is indicated for postoperative pain and is available on prescription only. However, because parecoxib sodium is not an FDA Orange Book drug, its marketing status is not currently curated and the *availability_type* is assigned as unknown.
-2 = A drug that has been withdrawn from the market for safety reasons for all medicinal products within a specified region of the world. This category is assigned if the drug has been manually curated in ChEMBL as Withdrawn (i.e., *molecule_dictionary.withdrawn_flag* = 1) and the marketing status is discontinued (for an FDA Orange Book drug) or the marketing status is unknown (where it is not an FDA Orange Book drug). Note that a drug can have marketing status of prescription and/or over-the-counter availability for one world region and be withdrawn for a different region of the world. For example ketoconazole (CHEMBL157101) is available as an FDA prescription drug (within the United States where it carries a black box warning for cardiotoxicity and hepatotoxicity^95^) but is manually curated as Withdrawn from Guatemala and Madagascar for drug-induced liver injury.^96^
- NULL = A research compound (i.e., not a drug or clinical candidate drug).

## Therapeutic Flag

The *therapeutic_flag* shows that an approved drug has a therapeutic application and is not solely indicated as a diagnostic agent (in the *molecule_dictionary* table, where 1 = yes and 0 is the default value). The *therapeutic_flag* is assigned unless the indication paragraph of the drug label contains phrases like: diagnostic agent, imaging agent, contrast agent, irrigating solution, cleansing, detection, gastrointestinal lavage, disclosing agent, sedation, echocardiograph, ultrasonograph, tomograph, etc. The *therapeutic_flag* is assigned by regular expression pattern match to the text describing the indication, followed by manual inspection and curation if required. For example, panitumumab (CHEMBL1201827) is indicated for colorectal neoplasms by the FDA^97^ and EMA,^98^ and has therapeutic_flag = 1 assigned. By contrast, Indium In-111 pentetreotide (CHEMBL1200642) is a radioactive diagnostic agent and has *therapeutic_flag* = 0 because it is used in gamma imaging (scintigraphy) to identify neuroendocrine tumors^99^ and not as a therapeutic treatment.

## First Approval

The *first_approval* field is the earliest known approval year for the drug (in the *molecule_dictionary* table, where NULL is the default value, Figure 9a). The earliest year of approval is curated for drugs that have been approved for human use (and not veterinary medicines, for example) and is routinely extracted for FDA New Drug Approvals and EMA drugs. For some drugs, the approval year has been manually curated for drugs from other regulatory agencies that are not routinely incorporated into ChEMBL such as PMDA.^77^ In cases where the drug has been approved, and later withdrawn, and subsequently reapproved, then the earliest year of approval is assigned. For example, fenfluramine hydrochloride (CHEMBL2106217) was approved as an antidepressant in France in 1963 and in the United States in 1973,^100^ but it was withdrawn from the United States in 1997 for safety concerns over valvular heart disease.^101^ More recently, it was reapproved in the United States in 2020 for the treatment of seizures associated with Dravet syndrome but carries a black box warning for valvular heart disease and pulmonary arterial hypertension.^102^ As a result, 1963 is assigned as the *first_approval* year for fenfluramine hydrochloride.

**Figure 9:**
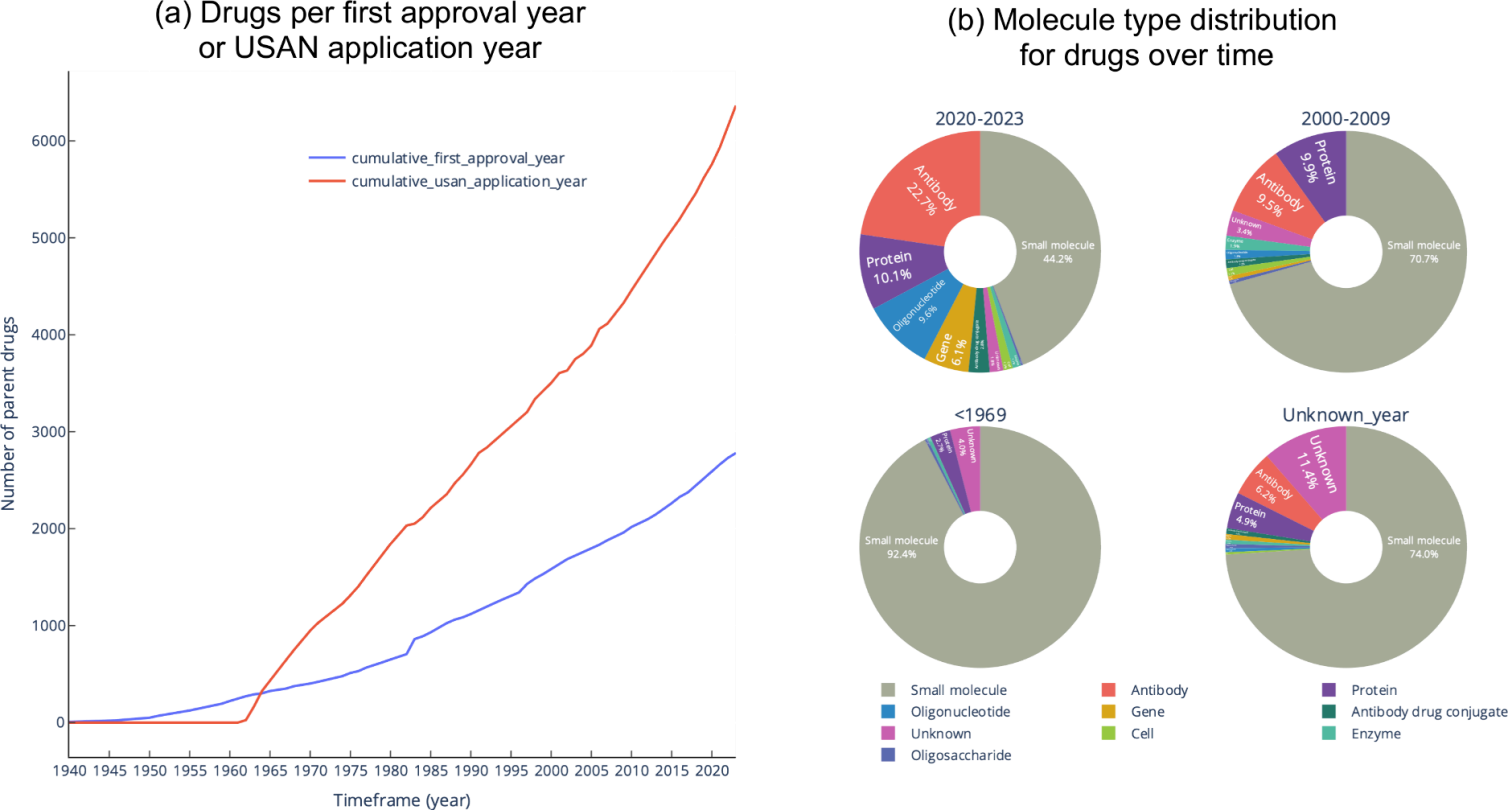
(a) Earliest year of approval or USAN application year for ChEMBL 35. The plot shows the cumulative count of distinct parent drugs between the years 1939 and 2023. (b) The distribution of molecule type as a function of the year of approval or the USAN application year. The plot shows the molecule type for the earlier of USAN application year or first approval year for each parent drug. The data are shown for selected time periods (top row and bottom left pie chart), and for drugs where there is no USAN application year nor first approval year assigned (“unknown” year in bottom right pie chart). Note that a parent drug is assigned the same molecule type as other drug forms within each compound family.

## USAN Year

The *usan_year* is the earliest known year in which the application for a USAN was granted (in the *molecule_dictionary* table, where NULL is the default value, Figure 9a). The year is routinely extracted for all new USAN name applications. Because the USAN name application is granted during the clinical development phase, the *usan_year* is checked against the *first_approval* year (which should have a later date) and any discrepancies are manually checked and curated.

## USAN Stem

For USAN compounds, the list of name stems represents common stems for which chemical and/or pharmacological parameters have been established.^103^ The list of USAN stems^103^ is updated in the *usan_stems* table, with the mapped stem assigned in *usan_stem*, *usan_substem* and *usan_stem_definition* fields in the *molecule_dictionary* table. The use of standard stems (prefix/infix/suffix) in the names of new USAN compounds means that similar compounds maintain a common “family” name that provides immediate recognition. For example, teriparatide (CHEMBL525610) has the USAN stem “-tide” which denotes that it is a peptide, specifically a parathyroid hormone related peptide.

## Orphan

The designation of orphan status means that a compound is intended for use against a rare condition. Currently, the *orphan* field is only applied to EMA compounds (in the *molecule_dictionary* table where 1 = yes, 0 = no, −1 = preclinical compound i.e., not a drug).

## Veterinary

The *veterinary* field indicates whether the compound is known to have a veterinary product and is planned to be introduced for ChEMBL 36 for EMA compounds only (in the *molecule_dictionary* table where 1 = yes, 0 = default value). Note that a veterinary product may also be a medicinal product for human use.

## Cross References

Any compound in ChEMBL with an InChI representation of a chemical structure (drug, clinical candidate drug or research compound) can be cross-referenced against an identical chemical structure in a different database using UniChem (www.ebi.ac.uk/unichem), our database of pointers between chemical structures. These cross-references are available via the web interface compound report card under the heading “UniChem Cross References”. For example, bentiromide (CHEMBL1200368) is cross-referenced against 20 other databases, including the ChEBI and SureChEMBL chemical biology resources from EMBL-EBI.

Additional cross-references for FDA and EMA drugs, and USAN and INN clinical candidate applications are captured in the web interface compound report card under the heading “Cross References”. Internally, these cross-references are captured in the *compound_xref* table and *xref_source* table, which is planned to be made publicly available for a future ChEMBL release. These cross-references include (i) compounds such as biotherapeutics that do not have a chemical structure and therefore are not included in UniChem. For example, the antibody, eculizumab CHEMBL1201828 has a cross-reference to its EMA medicinal product called Soliris,^104^ and (ii) pointers to drug labels or clinical candidate applications where the chemical structure is not readily available as an InChI representation and therefore may not be included in UniChem.

Much progress has been made across the chemical sciences community to improve FAIRness with, for example, chemical structure standardization (see Compound Registration), chemical identifiers (like InChI, see Molecule Structure) and the use of ontologies and controlled vocabularies (see e.g., Indication). However, crucial aspects for comprehensive computational analyses such as data interoperability between independent resources are not always completely straightforward and therefore ChEMBL is continually improving its coverage of cross-references and the transparency of its data provenance.

## Data Quality Assessment

An assessment of the quality of the drug and clinical candidate drug data in ChEMBL is not easy to quantify because there is no single, absolute measure of data quality, although good indicators include data accuracy, completeness, uniqueness, consistency, timeliness and validity.^105^ The following paragraphs attempt to assess the quality of the data, using measurable metrics quantified during the drug and clinical candidate drug data extraction, curation and integration processes.

### Data Accuracy

Data are accurate when it reflects reality. High accuracy data leads to correct reporting, confident decision-making and a trusted database. There were 8 drug-related ChEMBL help-desk enquiries in the first six months of 2025, out of 201 help-desk tickets directed to the wider ChEMBL (4%). Over the same period, there were 1091 downloads of the ChEMBL 35 database, 217,000,000 requests to ChEMBL API end points by 170,000 unique hosts, and 2.7 million Web site page views. The drug-related help-desk enquiries ranged from advice on the curation of indications for single ingredient and combination ingredient medicinal products, advice for extracting targets for approved drug and clinical candidate drugs, advice on WHO ATC drugs and INN clinical candidate drugs, a query about drug or clinical candidate drug mapping between ChEMBL and an external resource, and data feedback on one research compound and one drug that were mapped to the same synonym, and one clinical candidate drug that required a correction to its chemical structure. These questions, combined with other interactions that our team have with the community of ChEMBL data users, demonstrate the need for transparency in the drug and clinical candidate drug extraction, curation, and integration processes. However, the relatively limited number of drug or clinical candidate drug-related questions via the ChEMBL help-desk or elsewhere shows that our data users are typically happy with the data quality that is provided, especially given the large number of data users that accessed ChEMBL data over the same time period. Internally, GitHub is used to track individual drug and clinical candidate drug issues that are noticed by the team during curation, integration or public engagement (such as delivering ChEMBL training courses). For the drug update for ChEMBL 34, there were 133 issues raised, of which 120 issues were closed during the same time period. These statistics show that significantly more issues are raised internally than via the external ChEMBL help-desk and demonstrates that our curation team is adept at identifying and correcting drug data to maintain its overall accuracy.

### Completeness

Data are considered complete when all the data required for a particular use is present and available to be used. In terms of compound coverage, the key sources of approved drug information from the FDA and EMA regulatory agencies are captured. Although there are many other national or continent-wide regulatory agencies (e.g., Japan’s Pharmaceuticals and Medical Devices Agency,^77^ Health Canada,^106^ Australia’s Therapeutic Goods Administration,^107^ or the newly created African Medicines Agency^108^), many of their drugs will have been captured by the inclusion of the FDA and EMA sources of approved medicinal products. For ChEMBL 34, of the 2334 parent drugs with an FDA regulatory source, only 193 parent drugs (8%) were added when the EMA source of approved drugs was included for the first time in ChEMBL. This shows that the approved drug coverage is good. For clinical candidate drugs, ChEMBL has comprehensive coverage because it captures interventions from ClinicalTrials.gov (8,875 drug forms) which is a widely used, worldwide source of clinical trials, as well as the international source of clinical candidate name applications (INN, 812 drug forms) and United States (USAN) name applications (12,590 drug forms). In terms of associated drug information, our existing processes capture a comprehensive range of data for drug indications, mechanisms of action, warnings and drug properties such as maximum phase of development, year of approval or USAN year, molecule type, chirality, first-in-class, route of administration, orphan status, veterinary status. Capturing additional drug forms from other sources and their associated information would be desirable but needs to be balanced against the significant practical effort required to extract wider information from sources that are typically unstructured, and to keep this information up-to-date on a regular basis.

### Uniqueness

This measures the number of duplicates. Data are unique if they appears only once in a data set. The compound registration process prevents the duplication of an identical chemical structure (see Compound Registration). However, there are edge cases where the incoming chemical structure has inconsistent stereochemistry, or a different drug form, but has the same synonym or trade name as a different compound, for example. These types of inconsistency are manually curated, and for ChEMBL 34, 131 existing drugs or clinical candidate drugs had their chemical structure manually amended out of 17,501 drug forms in ChEMBL 34 (0.75%). For the forthcoming ChEMBL 36 release, 139 chemical structures for drugs or clinical candidate drugs will have been manually amended. The relatively small number of manually corrected chemical structures across the drug and clinical candidate space in ChEMBL show the high-quality and robust nature of our data extraction, curation and integration processes. If there is no chemical structure (as in the case of biotherapeutic compounds) or the original data source contains no chemical structural information (e.g., intervention names given by ClinicalTrials.gov or the WHO ATC classification), then the name, synonym or trade name must be matched to an existing drug or clinical candidate name within ChEMBL. Therefore, ChEMBL has an ongoing curation effort to minimize duplicate drug or clinical candidate drug name data. For example, the number of duplicate pairs of pref_names for an individual drug form and its corresponding parent drug form has been reduced from 243 pairs for ChEMBL 32 to 176 pairs for ChEMBL 35. Similarly, the number of synonyms (or trade names) mapped to more than one drug form has been reduced from 2043 synonyms for ChEMBL 32 to 1843 synonyms for ChEMBL 35. In the forthcoming ChEMBL 36 release, these statistics will be further reduced to 39 duplicate pref_name pairs remaining for drug and clinical candidate drug data, and 692 synonyms mapped to more than one drug form.

### Consistency

This is achieved when data values do not conflict with other values within a record or across different data sets. Following on from the previous measure of uniqueness, our curation and integration processes identify and manually correct instances where incoming drug form is inconsistent with an existing drug form and its name, synonym or trade name. For instance, as explained in the Data Sources section, the high-level EMA summary information may display the parent drug form although the detail contained within the medicinal product documentation shows the individual (salt) drug form. 78 out of 882 drugs with an EMA source (9%) had their chemical structure or pref_name amended based on this type of inconsistency for ChEMBL 34. The detailed nature of our drug and clinical candidate drug checks that identify these seemingly hidden inconsistencies demonstrates the care and attention that is required to produce a fully coherent overall result.

### Timeliness

This indicates whether the data are available when expected and needed. For each drug release of ChEMBL, all drug and clinical candidate data for all sources are updated. This means that drug and clinical candidate drug data may be added, updated or removed for each source, as appropriate. For example, the WHO ATC contains 3454 drugs for ChEMBL 34. Of these, 96 (parent) drugs were added when compared to ChEMBL 32, and 28 (parent) drugs were removed from this source (although note that the drug may remain under a different drug or clinical candidate source). Therefore, 124 out of 3454 drugs with a WHO ATC source (4%) were amended. Clarity of the date at which the data were extracted from each source is an important aspect of timeliness and progress has been made toward timestamping each source of drug and clinical candidate data. For example, the extraction dates for our clinical trials and black box warning pipelines were included in the ChEMBL 34 release notes and this will be extended to the other drug and clinical candidate drug sources for the forthcoming ChEMBL 36 release.

### Validity

This is defined as the extent to which the data conforms to the expected format, type, and range. Our curation processes include rules to standardize drug and clinical candidate drug pref_names (see Preferred Name, Synonym and Trade Name Curation). The pref_name was amended for 158 out of 17,501 drugs or clinical candidate drugs for ChEMBL 34 (1%). Mapping of ontology terms for indications are subject to limits whereby only certain branches of the MeSH tree are used (see Indication). This approach restricts the range of ontology identifiers to focus on disease or diagnostic indications only (e.g., the drug treats a bacterial infection), and excludes ontological identifiers for the type of drug (e.g., the drug is an antibacterial compound). For each drug release of ChEMBL, the MeSH or EFO identifiers are amended to update any identifiers that have been downgraded, merged or have had their granularity amended by the ontology provided. For ChEMBL 34, there were 267 obsolete EFO identifiers that were remapped (out of 2408 EFO identifiers, 11%) and one obsolete MeSH identifier that was remapped (out of 2089 MeSH identifiers, 0.05%).

## Challenges

There are many challenges to curate, and regularly update, drug data while at the same time maintaining the high-quality accurate information that underpins the ChEMBL ethos. Some of the key challenges are described below.

### Disparate Types of Data Structure

Information is extracted from a wide range of data structures. Some of these may be easier to ingest semiautomatically while others require higher levels of manual checking and curation to maintain accuracy. Types of data structure may include (i) other databases (e.g., Orange Book text files), (ii) web pages (e.g., FDA New Drugs, FDA Biological License Applications), (iii) API programmatic access (e.g., ClinicalTrials.gov), (iv) excel spreadsheets (e.g., USAN stems, EMA), (v) pdf documents (e.g., EMA, USAN, INN, drug labels for indications, mechanisms, prodrugs), (vi) scientific literature papers (e.g., prodrugs and their pharmacologically active ingredients, withdrawn drugs, mechanisms). Each incoming data structure requires a different approach and appropriate scripts or notebooks to accurately ingest information, flag up inconsistencies and perform curation as required.

### Inconsistencies between Different Data Sources for the Same Compound

Recently, our curation effort has focused on maintaining accuracy between drug data shown in ChEMBL and its original source(s). This has been a particular issue for mapping an individual drug form versus the parent drug form to its original source information, with this level of curation not consistently applied for legacy ChEMBL data. Many of the drug sources have high level summaries that sometimes show the parent drug form (which is usually the INN drug form) although the individual drug form (salt/solvate/isotope) is described within underlying documents such as the medicinal product label or similar. As a result, care has been taken to map recent drug information to the appropriate drug form and, when inconsistencies between the drug form given in different drug sources are spotted, to manually check and remap to the appropriate individual drug form. Additional sources of evidence such as the trade name may also be inspected because the trade name is usually associated with only one individual drug form.

### Absence of Definitive Information to Uniquely Identify a Compound

If a chemical structure is not available, for example it is a biotherapeutic compound or the chemical structure is not drawn within the source information (e.g., ClinicalTrials.gov, WHO ATC) then the compound must be mapped by name, synonym or trade name. A name is not a unique identifier for a compound. For example, e.g., a research code or synonym may be used for more than one chemical structure, or a chemical structure may be described by more than one chemical name. For example, the trade name “anavex” and research code “anavex 2–73” are shown in the USAN source information^109,110^ for both the individual drug form blarcamesine hydrochloride (CHEMBL4594269) and its parent drug form blarcamesine (CHEMBL4297224). This type of issue causes challenges when mapping incoming drugs to existing compounds and an ongoing curation focus has been to correct existing duplicate names, synonyms and trade names, and put checks in place that minimize incoming name issues.

### Long-Term, Continual Improvement, and Maintenance of Drug Data, Code, and Database Infrastructure

The drug and clinical candidate drug data are an integral part of ChEMBL, a trusted resource that is intended to be maintained for decades. EMBL-EBI commits to long-term data preservation,^111^ and ChEMBL is supported by core and grant-funding (see Acknowledgment). Given our lean and finite amount of human resource available, this presents a real challenge to continually improve and maintain (i) up-to-date drug and clinical candidate drug data, (ii) a sustainable code base, and (iii) database infrastructure. This challenge is especially noteworthy at the current time as data users perform drug discovery at scale using ChEMBL data. Therefore, ChEMBL is exploring methods to scale up and increase the efficiency of its drug data and other processes while at the same time maintaining the highest quality output that requires human-in-the-loop intervention.

## Access to the Curated Drug Data

ChEMBL (www.ebi.ac.uk/chembl) provides a number of mechanisms to search and retrieve relevant drug information. The data are provided under a CC-BY-SA 3.0 license. Relevant web interface pages for accessing drug data include:

- Data for an individual drug form (www.ebi.ac.uk/chembl/explore/compounds). Note that using *max_phase* is not equal to NULL captures relevant drug information only.
- Data for a parent drug form (www.ebi.ac.uk/chembl/explore/drugs). This currently shows data aggregated onto the parent drug form.
- Drug indications (www.ebi.ac.uk/chembl/explore/drug_indications)
- Drug mechanisms (www.ebi.ac.uk/chembl/explore/drug_mechanisms)
- Drug warnings (www.ebi.ac.uk/chembl/explore/drug_warnings)

In addition, the drug data are available through an API using multiple distinct web service end points (https://www.ebi.ac.uk/chembl/api/data/docs). The drug data is available for download in multiple formats from our FTP site (https://chembl.gitbook.io/chembl-interface-documentation/downloads). ChEMBL provides many different types of training material and documentation to assist data users. This includes multiple introductory and advanced webinars on data and using the API, a quick tour, FAQs, a blog and tailored workshops (see https://chembl.gitbook.io/chembl-interface-documentation/training-material). A ChEMBL python client is also available. ChEMBL data users are encouraged to make use of these resources and to direct any feedback, questions or comments to the helpdesk (chembl-help@ebi.ac.uk). An example SQL query to capture all data with a drug source (i.e., compounds with *src_id*: 8, CANDIDATES; 9, FDA_ORANGE_BOOK; 12, FDA_NEW_DRUGS; 13, USAN; 36, WITHDRAWN; 41, ATC; 42, BNF; 53, PRODRUG_ACTIVE; 63, INN; 66, EMA) is:

select * from chembl_35.molecule_dictionary md where md.molregno in (select molregno from chembl_35.compound_records where src_id in (8, 9, 12, 13, 36, 41, 42, 53, 63, 66)

## Technical Process

The overall process to update the drug data is a combination data extraction from multiple sources, mapping to existing data followed by data integration into “Drugbase”, a relational Oracle database. As part of these processes, any obsolete information is downgraded, and many checks are performed before, during and after the data loading. There are around 50 (mainly python) scripts and notebooks that take up to six months to run. These scripts and notebooks automate our processes as far as possible but also flag up any issues that need manual inspection and correction before the data are loaded. This means that a larger proportion of time is spent performing manual curation to correct inconsistencies and prevent issues rather than loading data. When our team is happy that “Drugbase” is internally consistent and passes all checks, the data are migrated to the ChEMBL database before release to the public. Any drug-related feedback raised by our data users via the ChEMBL helpdesk (chembl-help@ebi.ac.uk) is also considered and data are corrected as required.

There has been a significant drive to improve the sustainability of our internal code base, documentation and working practices over the past few years. This included maintaining and updating python environments and packages, implementing version control for all scripts, notebooks and processes, vastly improving internal documentation for the code base and curation and integration processes, providing and clarifying external documentation (such as this manuscript, FAQs, blog posts), improving database transparency by using common shared internal database areas for all curation and data integration work, dropping hundreds of redundant internal database tables, adding or clarifying internal database metadata, changing working practices so each process is run by more than one team member resulting in more discussion, improved clarity of the process and better documentation. Although big steps forward have been achieved, continual improvement for the drug and clinical candidate drug processes is ongoing.

Despite our extensive set of checks, caveats and omissions may be present in the original sources of data and our processes depend on the accuracy of the original documents and our data extraction, curation and integration processes. It is worthwhile noting that some information may not be available for legacy data and therefore cannot be accurately curated. ChEMBL provides many web URL references to maintain data transparency. In addition, it is recommended that data users perform data checks before using any data in ChEMBL and that users are responsible for data cleanup, understanding of their data sets and appropriate interpretation of results.

## Conclusions

The paper presents the state-of-the-art processes to curate and integrate the high-quality drug and clinical candidate drug data in ChEMBL. The drug curation processes have been developed over more than 15 years and this is the first time that they have been published. The inclusion of high-quality, well-curated drug data within the structured, open-access framework of the ChEMBL bioactivity database provides a valuable asset to the chemical biology community as it moves toward wider use of artificial intelligence in drug discovery and its inherent dependence on high-quality data.

## ‡

The Francis Crick Institute, 1 Midland Road, London NW1 1AT, U.K

## §

LifeArc Accelerator Building, Open Innovation Campus, Stevenage, London SG1 2FX, U.K.

## Abbreviations

BNF: British National Formulary
EMA: European Medicines Agency
FDA: Federal Drug Administration
GSRS: Global Substance Registration System
INN: International Nonproprietary Names
MHRA: Medicines and Healthcare Regulatory Agency
PMDA: Pharmaceuticals and Medical Devices Agency
USAN: United States Adopted Names
WHO ATC: World Health Organisation Anatomical Therapeutic Classification
